# Nomophobia in Nursing Students: Psychological, Academic, and Clinical Impacts—An Integrative Review

**DOI:** 10.3390/healthcare14070830

**Published:** 2026-03-24

**Authors:** Assunta Guillari, Andrea Chirico, Chiara Palazzo, Maurizio Di Martino, Francesco Cristiano, Salvatore Suarato, Teresa Rea, Vincenza Giordano

**Affiliations:** 1 Clinical Research Center DEMeTra, Department of Translational Medical Sciences, University of Naples “Federico II”, 80138 Naples, Italy; assunta.guillari@unina.it; 2 Department of Developmental and Social Psychology, University of Rome “La Sapienza”, 00185 Rome, Italy; andrea.chirico@uniroma1.it; 3 Department of Biomedicine and Prevention, University of Rome “Tor Vergata”, 00133 Rome, Italy; 4 Division of Haematopoietic Stem Cell Transplantation and Cellular Therapies, Azienda Ospedaliera di Rilievo Nazionale Santobono-Pausilipon, 80122 Naples, Italy; 5 Department of Translational Medical Sciences, University of Naples “Federico II”, 80138 Naples, Italy; maurizio.dimartino@unina.it; 6 Master’s Degree Program in Nursing and Midwifery Sciences, Department of Neuroscience, Reproductive and Odontostomatological Sciences, University of Naples “Federico II”, 80138 Naples, Italy; francesco.cristiano4@studenti.unina.it (F.C.); sa.suarato@studenti.unina.it (S.S.); 7 Department of Public Health, University of Naples “Federico II”, 80138 Naples, Italy; teresa.rea@unina.it (T.R.); enza-giordano@hotmail.com (V.G.)

**Keywords:** nomophobia, nursing students, smartphone use, digital dependence, mental health, academic performance, integrative review, clinical decision-making, digital well-being

## Abstract

**Highlights:**

**What are the main findings?**

- Nomophobia among nursing students is highly prevalent globally and is consistently associated with anxiety, depression, sleep disturbance, fear of missing out (FoMO), and reduced academic performance.
- Integrative evidence identifies excessive smartphone use, emotional dysregulation, and low resilience as central mechanisms underlying problematic digital behaviors in nursing education.

**What are the implications of the main findings?**

- Nomophobia represents a relevant digital health and mental health issue in nursing education, with potential consequences for clinical decision-making, professional competence, and patient safety.
- Integrating digital well-being, emotional regulation, and resilience-focused interventions into nursing curricula may mitigate maladaptive smartphone use and support safer, more competent future healthcare professionals.

**Abstract:**

**Background/Objectives**: Nomophobia, the irrational fear of being without a mobile phone, is increasingly prevalent among university students and has emerged as a concerning form of digital dependence. Among nursing students, this condition is particularly relevant due to the emotional demands and cognitive challenges of healthcare education. Nomophobia has been linked with adverse psychological outcomes, sleep disturbances, and impaired academic and clinical performance. However, existing evidence remains fragmented and lacks an integrated conceptual synthesis. This review aimed to synthesize current evidence on the prevalence, correlates, and outcomes of nomophobia among nursing students. **Methods**: An integrative review was conducted following Whittemore and Knafl’s methodology and PRISMA guidelines. A systematic search was performed in PubMed, CINAHL, PsycINFO, PsycArticles, and Medline (between 2015 and 2025), supplemented by Google Scholar. Cross-sectional studies and literature focusing on nomophobia in nursing students were included. The primary studies and selected review articles were considered when no overlap with the included primary evidence was identified. Methodological quality appraisal was assessed using validated tools (QuADS and JBI). **Results**: Twenty-two studies were included (19 cross-sectional and 3 reviews). Four thematic areas emerged: prevalence and severity (50–90% moderate to severe); psychological correlates (anxiety, depression, stress, insomnia, alexithymia, fear of missing out); academic and cognitive outcomes (impaired performance, procrastination, reduced decision-making); and behavioural predictors (excessive smartphone use and emotional dysregulation). The Nomophobia Questionnaire (NMP-Q) was the most frequently used instrument. **Conclusions**: Nomophobia represents a relevant dimension of the mind–technology relationship in nursing education, with implications for students’ mental health, academic engagement, and clinical readiness. Addressing nomophobia may support healthier learning environments and contribute to the development of emotionally competent and safe future healthcare professionals. However, significant gaps remain, particularly regarding longitudinal evidence and intervention-based approaches.

## 1. Introduction

Over the last two decades, the use of smartphones has become an integral part of everyday life, playing a central role in communication, learning, and social networking. Younger generations, particularly university students in the health-related disciplines, are among the heaviest users of mobile technologies for both academic and personal purposes, including accessing educational resources, consulting bibliographic databases, participating in digital learning platforms, and maintaining social connections [1,2,3].

While constant connectivity provides important opportunities for information access and educational support, excessive or dysfunctional use of smartphones has also given rise to emerging forms of psychological discomfort, including nomophobia (no mobile phone phobia).

Nomophobia is defined as an irrational fear or clinically significant anxiety associated with being unable to access one’s smartphone, losing network connection, or being disconnected from digital social environments [4]. Initially considered a colloquial expression, this phenomenon is now the subject of growing scientific attention and is placed within the broader spectrum of behavioural addictions [5]. It may manifest through compulsive device checking, anticipatory anxiety when access is limited, and difficulties regulating emotions when disconnected from digital networks [6].

From an epidemiological perspective, nomophobia is particularly prevalent among adolescents and young adults. Prevalence estimates among university students range from 60% to 90%, depending on geographical context and measurement instruments [7,8,9]. For example, Dasgupta et al. (2017) [10] reported that more than 40% of medical and engineering students in India exhibited moderate levels of nomophobia.

Similarly, studies conducted in Europe, Latin America, and Asia suggest that over 70% of nursing students report moderate to severe levels of nomophobia [1,11].

The Nomophobia Questionnaire (NMP-Q) is the most widely used and validated psychometric instrument for assessing nomophobia due to its multidimensional structure and strong internal reliability [4,12]. Other instruments, such as the Mobile Phone Problematic Use Scale (MPPUS) [13] and the Mobile Phone Addiction Index (MPAI) [14], are sometimes used to assess broader constructs related to problematic smartphone use or addiction, although they do not specifically measure nomophobia. Several sociodemographic and psychological variables have been associated with higher nomophobia scores, including younger age, female gender, low self-esteem, social anxiety, and intensive social media use [5,6].

Nursing students may be particularly vulnerable to these dynamics due to their high exposure to digital learning environments and the demanding nature of their academic and clinical training [11]. Mobile devices are frequently used to access educational materials, communicate with teachers, share clinical experiences, and maintain peer support networks. However, excessive reliance on smartphones may also contribute to dysfunctional patterns of use and increase the risk of anxiety and depressive symptoms [7].

A growing body of research has documented the potential psychological consequences of nomophobia, including anxiety, stress, depression, insomnia, and psychosomatic symptoms [1,6,7]. At a cognitive level, nomophobia has been associated with difficulties in concentration, planning, and time management [2]. These patterns may contribute to emotional distress and reduced psychosocial well-being among students [5].

Some authors have particularly highlighted the correlation between nomophobia and academic burnout, indicating that constant connectivity and the pressure of online social interaction can lead to emotional exhaustion and demotivation [15]. However, research on the impact of nomophobia on academic performance remains inconclusive. While some studies have reported a negative association between nomophobia levels and academic performance [5], other evidence suggests that this relationship may be indirect or weak, highlighting the potential role of mediating psychological factors such as emotional regulation, stress, or related affective processes [16,17].

Indeed, problematic smartphone use has been associated with reduced sleep quality, increased distraction, and diminished cognitive efficiency among university students [7].

Recent evidence further highlights the high prevalence of nomophobia among nursing students. For example, Sadeghi et al. (2025) reported that all participants in their sample experienced at least mild nomophobia symptoms, with nearly one-fifth (19%) presenting severe levels [18]. The study also found a significant positive correlation between nomophobia and social anxiety, suggesting potential implications for students’ psychological well-being and social functioning during clinical training.

From a theoretical perspective, the study of nomophobia has evolved through the incorporation of various psychological constructs. One of the most relevant is Fear of Missing Out (FoMO), defined as the persistent fear of being excluded from rewarding social experiences [19]. FoMO has emerged as a significant predictor of nomophobia in several studies and may partially mediate the relationship between problematic smartphone use and academic burnout [7,16]. Additional psychological traits, including impulsivity, boredom proneness, emotional dysregulation, and alexithymia, have also been associated with the development and maintenance of nomophobia, highlighting its multifactorial nature [1,2,6,20]. Compulsive social media use represents another important behavioural component, reflecting the central role of digital interaction in the nomophobic experience [21].

The digitisation of healthcare education, further accelerated during the COVID-19 pandemic, has intensified students’ exposure to continuous connectivity and blurred the boundaries between academic, professional, and personal smartphone use [11,15,16]. In this context, understanding the potential impact of hyperconnectivity on the mental well-being of nursing students becomes particularly relevant. Beyond its potential impact on students’ mental health and academic functioning, nomophobia may also influence clinical learning processes and professional development. Emerging evidence suggests that high levels of smartphone dependence may be associated with reduced empathy, impaired clinical decision-making, and increased distraction in healthcare settings, potentially affecting professional behaviour and patient safety [22,23]. Despite the growing body of literature on nomophobia, existing evidence remains fragmented. Studies differ substantially in terms of methodological design, measurement instruments, and sampled populations, making it difficult to draw comprehensive conclusions.

Given these considerations, synthesizing the available evidence is essential to better understand the prevalence, determinants, and consequences of nomophobia in this population. An integrative review approach, as described by Whittemore and Knafl [24], is particularly suitable for examining complex and emerging phenomena, as it allows the integration of evidence derived from diverse methodological approaches and facilitates a broader conceptual interpretation of the literature.

Therefore, the aim of this integrative review is to synthesize the available evidence regarding nomophobia among nursing students by: (1) examining its prevalence and severity; (2) identifying associated sociodemographic and individual factors; (3) exploring its implications for mental health and well-being; (4) analyzing its potential impact on academic performance; (5) examining its relationships with FOMO, alexithymia, compulsive social media use and other psychological variables; and (6) discussing its implications for nursing education and clinical training.

## 2. Materials and Methods

This integrative review was conducted following the methodological approach proposed by Whittemore and Knafl [24], which allows for the inclusion, analysis, and critical synthesis of heterogeneous evidence. In the present review, primary empirical studies constituted the core of the synthesis, while review articles were screened to contextualize the topic and identify additional relevant primary studies, but were not included in the analytical synthesis of findings. This type of review is not limited to aggregating existing data but aims to reformulate and expand the knowledge base on a topic, generating theoretical and practical insights [25]. This approach is particularly suitable for the investigation of nomophobia, a multifactorial condition that intersects psychological, behavioural, academic, and professional domains, and for which the current literature is marked by variability in study design, population characteristics, and measurement tools.

The review adhered to the Preferred Reporting Items for Systematic Reviews and Meta-Analyses (PRISMA 2020) guidelines where applicable [26], and followed six iterative steps: (1) formulation of a focused research question; (2) development of a systematic search strategy; (3) application of inclusion and exclusion criteria; (4) critical appraisal of the methodological quality of included studies; (5) data extraction, thematic categorization, and narrative synthesis; and (6) integrative interpretation of findings across sources.

### 2.1. Search Strategies

A comprehensive literature search was conducted across five major databases: PubMed, CINAHL Complete, APA PsycINFO, APA PsycArticles, and Medline, including studies published between January 2015 and June 2025 and available in English. Additionally, Google Scholar was used as a supplementary search source to reduce database bias and identify potentially relevant peer-reviewed studies not indexed in the primary databases. In line with common practice when using Google Scholar in systematic and integrative reviews, screening was limited to the first 50 results sorted by relevance to balance sensitivity with feasibility and to reduce the inclusion of poorly indexed or non-peer-reviewed materials [27]. Only peer-reviewed studies meeting the predefined inclusion criteria were included in the final synthesis. The search terms were identified using the PEO framework (Population, Exposure, Outcome), which is suitable for qualitative and observational research questions [28,29]:

- Population (P): nursing students.
- Exposure (E): nomophobia, smartphone addiction, problematic mobile phone use.
- Outcome (O): effects on psychological well-being, mental health, academic performance, decision-making skills, and sleep quality.

The use of Boolean operators AND and OR allowed us to combine relevant MeSH terms and keywords to identify studies concerning nomophobia (including synonyms such as “smartphone addiction”, “problematic smartphone use”, “mobile phone dependence”) and the nursing student population (e.g., “nursing students”, “student nurses”). To fully reflect the objectives of the review, terms related to associated outcomes, such as academic performance, mental health and well-being, as well as clinical skills (e.g., “decision-making”, “clinical skills”, “concentration”, “attention”) were also included. These terms were applied in the Title/Abstract fields. The complete search strings, including all synonyms and related search fields, are shown in Table S1, together with the number of results obtained for each strategy. Preliminary versions of the strings were tested and optimized to maximize sensitivity while avoiding an excessive number of irrelevant results.

All searches were conducted independently by two reviewers (C.P. and A.G.), and the results were managed using Rayyan—Intelligent Systematic Review software to facilitate the selection process and reduce the risk of bias [30]. Conflicts during screening or quality appraisal were resolved through consensus meetings with a third reviewer, ensuring methodological rigour. Initial screening was performed on titles, with duplicates being eliminated. Any disagreements between reviewers were resolved through discussion and consensus.

### 2.2. Inclusion and Exclusion Criteria

Inclusion criteria:

- Studies focusing on undergraduate nursing students.
- Studies investigating nomophobia or closely related constructs (e.g., smartphone addiction, problematic smartphone use).
- Studies assessing at least one of the following outcomes: psychological well-being (e.g., stress, anxiety, depression), academic performance, cognitive functioning (e.g., concentration, attention, decision-making), or sleep quality.
- Empirical studies using quantitative designs (cross-sectional, correlational). Literature reviews were screened to contextualize the topic and identify additional primary studies, but were not included in the analytical synthesis of primary findings.
- Only peer-reviewed publications are eligible.

Exclusion criteria:

- Studies that do not disaggregate data for nursing students if other health-related populations are included.
- Editorials, commentaries, opinion papers, conference abstracts, or grey literature without peer review.
- Studies using qualitative designs without clear methodological reporting.
- Studies not available in full text or published outside the defined time frame (2015–2025).

### 2.3. Study Selection and Data Management

**Figure 1:**
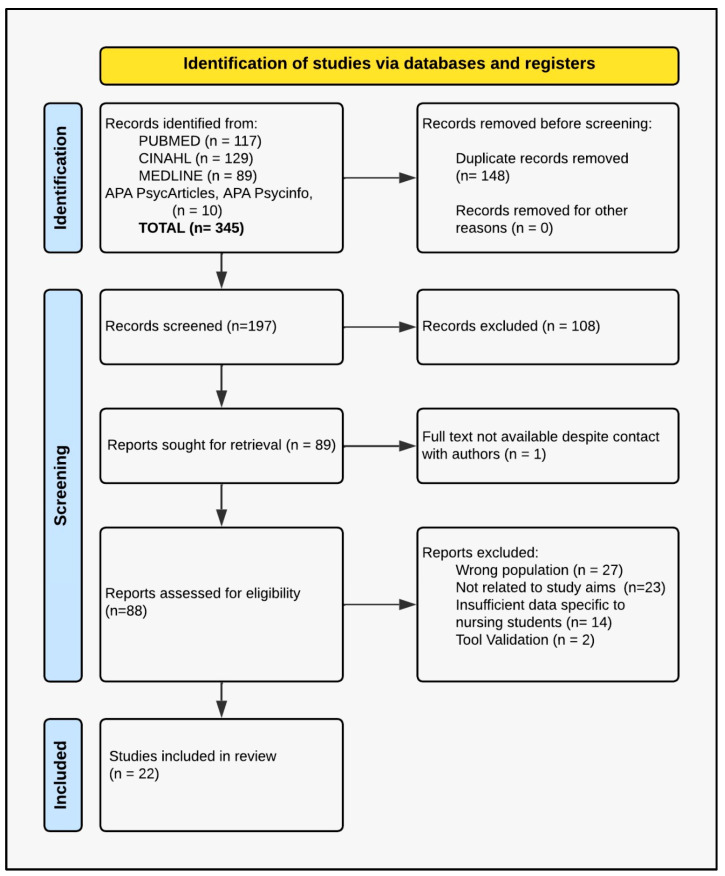
PRISMA 2020 flow diagram of the study selection process.

A total of 345 articles were identified, of which 88 were selected for full-text assessment. At this stage, 66 articles were excluded because they did not meet the inclusion criteria or due to duplication or overlap with already included primary studies. As a result, 22 articles were included in the final review. The entire process of identification, screening, eligibility, and inclusion was documented and represented graphically according to the PRISMA 2020 flowchart (Figure 1), adapted to the methodological context of integrative review, following the PRISMA guidelines [26]. To minimize double-counting, data from included reviews were used only for contextual triangulation and were not pooled with primary study outcomes. Duplication or overlap was checked by cross-matching the first author, year, country, and sample size; when overlaps were detected, only the primary study was retained in quantitative summaries.

### 2.4. Quality Appraisal

The methodological quality and risk of bias of the included studies were appraised according to the principles of integrative reviews, ensuring transparency and consistency [24,31]. Given their methodological diversity, appropriate validated tools were applied: the JBI Checklist for Analytical Cross-Sectional Studies [32] for cross-sectional designs (*n* = 19) and the QuADS tool [33] for qualitative and review-based studies (*n* = 3). For the JBI checklist, scores ranged from 0 to 8 and were classified as high (7–8), moderate (5–6), or low (<5). For the QuADS tool, the maximum score was 39, with thresholds of high (30–39), moderate (20–29), and low quality (<20). Quality thresholds for the JBI were author-defined solely to stratify confidence in the narrative synthesis, whereas QuADS scoring followed the itemization of Harrison et al. Two reviewers independently appraised study quality, and disagreements were resolved by consensus (percent agreement = 90%, Cohen’s κ = 0.82). Risk of bias was considered proportional to methodological quality. In quantitative studies, common sources of bias include sample selection, unadjusted confounders, and reliance on self-reported data. In qualitative and review studies, limitations were mainly insufficient theoretical justification, limited stakeholder involvement, and reduced analytic transparency. Findings from high-quality studies were regarded as robust, whereas evidence from moderate- or low-quality studies was interpreted with caution and contextualized through data triangulation. Detailed scores are provided in Tables S2a (QuADS) and S2b (JBI). Particular attention was given to potential sources of bias related to measurement heterogeneity, the predominance of cross-sectional designs, and the reliance on self-reported data.

### 2.5. Data Extraction and Synthesis

Data synthesis followed the integrative review process described by Whittemore and Knafl [24], involving data reduction, comparison, and thematic categorization of findings across studies. Results were then integrated through a narrative synthesis in order to identify recurring patterns and relationships across heterogeneous sources of evidence. Twenty-two studies investigating nomophobia and problematic smartphone use among nursing students were included. Nineteen primary studies were included in the analytical synthesis. Three additional literature reviews were screened to contextualize the topic and support the interpretation of findings. No qualitative, quasi-experimental, or experimental studies were identified. The geographical distribution of the evidence shows a prevalence of contributions from Turkey, followed by India, with further research coming from Iran, Saudi Arabia, Spain, Italy, Egypt, and the United States. This geographical diversity provides insight into the global distribution of research on nomophobia among nursing students. Detailed data extraction for the included studies is reported in the Supplementary Materials (Table S3 for review studies and Table S4 for quantitative studies).

## 3. Results

### 3.1. Overview of Included Studies

Narrative synthesis was used to integrate the findings, and conceptual triangulation was employed to cross-validate evidence from different methodological sources. Four main thematic areas were identified: (1) prevalence and severity of nomophobia, with significant levels of distress associated with dysfunctional smartphone use; (2) associations between nomophobia and psychological variables, highlighting relationships with anxiety, depression, stress, insomnia, alexithymia and fear of missing out (FoMO); (3) effects of excessive smartphone use on academic performance, attention, concentration and decision-making skills; (4) behavioural and psychological predictors of nomophobia. These themes correspond to the predefined objectives of the review and provide a structured synthesis of the available evidence. A concise overview of the included studies is provided in Table 1, summarizing authors, year, country, study design, sample, and principal findings.

### 3.2. Prevalence and Severity of Nomophobia in Nursing Students

Nomophobia is highly prevalent among nursing students, with most studies reporting moderate to severe levels of smartphone dependence and associated psychological discomfort. In a cross-sectional study, Gaber-Hamzaa et al. reported that 79.3% of students experienced moderate nomophobia and 16.8% severe levels, with a mean score of 91.2 ± 22.8 on the Nomophobia Questionnaire (NMP-Q) [43]. Similarly, Conte et al. found that 66.5% of Italian nursing students scored in the moderate-to-high range, while only 9% exhibited mild symptoms [40]. In the Turkish context, Bilgiç reported that 92.5% of students had moderate or severe levels [39]. Çobanoğlu et al. observed that 85.9% of students were in the moderate range, and only 2.1% were symptom-free [6]. Kargın also reported high average scores on both the NMP-Q and FoMO scales, consistent with elevated nomophobia severity [45]. In Iran, Janatolmakan et al. found that 64.4% of students were in the moderate range and 12.2% in the severe range [11]. Indian studies show some variability: Anand et al. found that only 2% of students had severe nomophobia, but over 30% exhibited behavioural indicators consistent with moderate risk [38]. Jose et al. reported a mean NMP-Q score of 79.3 (SD = 23.1), indicating a relatively high average severity within the sample [44]. Similarly, Yigit reported elevated nomophobia scores in association with psychological distress [7]. These patterns are reinforced by the findings of three literature reviews. The scoping review by Zhou et al., which analyzed 39 studies from 15 countries, reported prevalence estimates ranging from 19% to 72%, with a central tendency between 40% and 50%, underlining both variability and widespread occurrence [36]. The review by Ramjan et al., which included both qualitative and quantitative studies, identified consistent evidence of problematic mobile phone use and digital dependency among nursing students, particularly in young female populations and frequent users [35]. Finally, Osorio-Molina et al., through meta-analytic synthesis, highlighted moderate to high levels of smartphone addiction and associated negative psychological and academic outcomes among nursing students [34].

Across the 19 primary studies, the reported prevalence of moderate to severe nomophobia showed a median estimate of approximately 72% (IQR: 60–84%). This value should be interpreted as a descriptive summary of the included studies rather than a pooled estimate, given the heterogeneity in study design, measurement instruments, and sample characteristics.

In contrast, the three included reviews (two scoping and one integrative) reported wider prevalence ranges (19–72%), reflecting methodological and cultural heterogeneity. Taken together, both primary and review-level evidence converge in demonstrating that nomophobia is a widespread and persistent phenomenon among nursing students worldwide.

### 3.3. Psychological Correlates of Nomophobia in Nursing Students

The included studies consistently demonstrate that nomophobia is associated with a range of psychological variables, most notably depression, anxiety, stress, psychological resilience, fear of missing out (FoMO), alexithymia, psychological alienation, and psychological resilience. Psychological distress was the most consistently associated domain. In a study of 544 Turkish nursing students, Yigit found moderate, positive, and statistically significant correlations between nomophobia and depression (r = 0.456), anxiety (r = 0.402), and stress (r = 0.498) using the DASS-21 (*p* < 0.001 for all) [7]. Similarly, Conte et al., using the HADS scale, reported correlations of r = 0.579 for anxiety and r = 0.436 for depression among Italian nursing students (*p* < 0.01) [40]. Akbari et al. in Iran reported weaker but still significant correlations between nomophobia and anxiety (r = 0.39) and depression (r = 0.31) [50]. Psychological resilience was inversely associated with nomophobia in Cobanoglu et al., who found a moderate negative correlation (r = −0.348, *p* < 0.001) using the CD-RISC, indicating that students with higher nomophobia levels had reduced adaptive coping capacity. Fear of missing out (FoMO) was addressed in multiple studies [6]. Tuna et al. reported that FoMO significantly predicted nomophobia, explaining 18.5% of its variance (*p* < 0.001) [16]. Kargın demonstrated that students with high FoMO scores also exhibited significantly elevated levels of nomophobia, reinforcing the conceptual and behavioural link between the two constructs [45]. Alexithymia, or the inability to identify and express emotions, was examined by Akın and Durmaz, who reported that 28% of nursing students scored above the clinical threshold on the TAS-20 [37]. Nomophobia scores were positively correlated with alexithymia total scores (*p* < 0.05), particularly in the subdomains of difficulty identifying and describing feelings, suggesting emotional regulation difficulties in students with high mobile phone dependence. Psychological alienation was assessed by Gaber-Hamzaa et al., who found significantly higher alienation scores among students with NMP-Q scores above 85 (*p* < 0.01), implying a relationship between nomophobia and perceived social disconnection [43]. The included reviews supported and expanded these findings. Zhou et al., in a scoping review of 39 studies across 15 countries, consistently reported associations between nomophobia and anxiety, depression, perceived stress, and FoMO, although the authors cautioned that heterogeneity in assessment tools limited comparability [36]. Ramjan et al. emphasized the presence of psychological distress and emotional vulnerability among nursing students, including stress, digital dependence, and sleep disturbance [35]. Osorio-Molina et al. confirmed moderate to high levels of psychological risk linked to smartphone use, especially in relation to anxiety and depression [34]. In sum, these results indicate that nomophobia in nursing students is not an isolated phenomenon but is systematically associated with a broader profile of emotional and psychological dysregulation. These associations are consistent across diverse geographic, cultural, and methodological contexts and underline the need for preventive strategies and psychosocial interventions within nursing education. While these psychological associations offer insight into the profile of students affected by nomophobia, the following section addresses how this condition may impact their academic and cognitive functioning.

### 3.4. Effects of Excessive Smartphone Use on Academic Performance, Attention, Concentration, and Decision-Making Skills

Evidence from the included studies suggests that excessive smartphone use and nomophobia are associated with lower academic performance, reduced attention regulation, decreased concentration, and difficulties in decision-making among nursing students.

Academic performance and achievement were negatively associated with problematic smartphone use in several studies. Jose et al., in a cross-sectional study of Indian nursing students, found that students with higher levels of problematic mobile phone use had significantly lower academic scores (*p* < 0.05) [44]. The study utilized self-reported academic grades and the Mobile Phone Problematic Use Scale, and emphasized the discrepancy between perceived and actual academic achievement. Bilgiç et al. reported that students who self-identified as addicted to smartphones were more likely to report deteriorating study habits, difficulty completing academic tasks, and increased mental fatigue [39]. Conte et al. similarly noted that nomophobia was associated with reduced time allocated to focused study and poorer academic performance during exam periods [40]. The integrative review by Osorio-Molina et al. highlighted negative effects on learning performance, concentration, and increased procrastination and disorganization of academic tasks in nursing students [34]. Attention and concentration difficulties were consistently observed. In the study by Çobanoğlu et al., students with high nomophobia scores reported significantly greater subjective distraction during lectures and while studying [6]. Kargın et al. noted that FoMO and internet addiction, both strongly associated with nomophobia, correlated with diminished sustained attention and increased task-switching tendencies [45]. Tuna et al. identified a predictive relationship between FoMO and nomophobia, and discussed its implications for academic distraction and task avoidance, specifically linking higher nomophobia with increased tendencies toward academic procrastination [16]. Conte et al. also reported that students experiencing higher levels of nomophobia were more prone to delay tasks and had difficulty maintaining study concentration, particularly under exam stress [40]. Decision-making skills and cognitive control were indirectly addressed in several sources. Akın and Durmaz noted that nursing interns with high nomophobia and alexithymia reported difficulties in clinical decision-making situations, particularly those requiring emotional interpretation or prioritization under pressure [37]. Zhou et al., in their scoping review, cited evidence from Tastan et al. indicating that high smartphone dependency in nursing students may interfere with decision-making and communication in clinical environments, potentially compromising patient care and safety [36,51].

The review by Zhou et al. supports and expands these findings, identifying reduced attention span, procrastination, impaired cognitive engagement, and weakened clinical reasoning as frequently reported consequences of nomophobia. However, the authors highlight that most of the reviewed studies employed cross-sectional designs and self-reported instruments, which limit the ability to infer causality [36]. Ramjan et al. also emphasized disruptions in study regulation and time management as recurrent issues, especially among junior nursing students [35]. Osorio-Molina et al. confirmed that problematic smartphone use is linked to poorer academic outcomes and difficulty maintaining concentration and decision-making under academic pressure [34]. Taken together, the data suggest that excessive smartphone use among nursing students may be associated with poorer academic functioning and reduced cognitive efficiency, with potential implications for professional preparedness and clinical competence. These findings underscore the importance of integrating digital health education and self-regulation strategies within nursing curricula. Building on these outcomes, the next section examines the behavioural and psychological factors that may predispose nursing students to nomophobia, shedding light on its underlying mechanisms

### 3.5. Behavioural and Psychological Predictors of Nomophobia

A range of behavioural and psychological factors emerged as significant predictors of nomophobia across the included studies. These include fear of missing out (FoMO), perceived stress, psychological distress (anxiety and depression), emotional dysregulation, psychological alienation, low resilience, patterns of social media use, and smartphone use duration. Several studies further identified mediators and moderators that clarify the relationships among these variables. FoMO and social media use were among the most robust behavioural predictors. Kargın et al., in a study involving 291 Turkish nursing students, found a strong correlation between FoMO and nomophobia (r = 0.715, *p* < 0.01), suggesting substantial conceptual and behavioural overlap [45]. Tuna et al. (2023) confirmed this relationship in a regression analysis, showing that FoMO significantly predicted nomophobia (β = 0.39, *p* < 0.001) [16]. Additionally, passive social media use emerged as a significant moderator, amplifying the effect of FoMO on nomophobia. Akin and Durmaz extended these findings by demonstrating that perceived stress partially mediated the relationship between FoMO and nomophobia, supporting a stress-based explanatory pathway [37]. Psychological distress, including symptoms of anxiety and depression, was consistently associated with nomophobia. In Yigit, moderate positive correlations were found between nomophobia and depression (r = 0.456), anxiety (r = 0.402), and stress (r = 0.498), all with *p* < 0.001, in a sample of 544 Turkish students [7]. Similarly, Akbari et al. found significant correlations between nomophobia and both anxiety (r = 0.39) and depression (r = 0.31) in an Iranian sample (*n* = 420) [50]. Jose et al. noted that perceived psychological burden, especially related to academic performance, was linked to increased smartphone dependency and nomophobia. Psychological alienation was another salient factor [44]. Gaber-Hamzaa et al. reported that students with high nomophobia (NMP-Q > 85) also scored significantly higher on the Psychological Alienation Scale (*p* < 0.01) [43]. This finding supports the hypothesis that perceived social disconnection contributes to problematic mobile phone use. Emotional dysregulation and alexithymia were discussed by Akin and Durmaz as contributing factors [37]. Although not always measured quantitatively, difficulties in identifying and expressing emotions were implicated as mechanisms that heighten emotional reliance on smartphones. This aligns with theoretical models of nomophobia as an emotion-focused maladaptive coping strategy. Resilience acted as a protective factor. In Çobanoğlu et al., a study of 850 students revealed a moderate inverse correlation between nomophobia and psychological resilience (r = −0.348, *p* < 0.001), suggesting that students with greater adaptive capacity are less prone to problematic smartphone use [6]. Smartphone use duration was a consistent predictor across multiple studies. Gaber-Hamzaa et al. reported that students using smartphones for more than 4 h daily had significantly higher nomophobia scores (*p* < 0.01) [43]. In Bilgiç, the majority of participants reported more than 5 h of daily use, and this high use was associated with elevated NMP-Q scores [39]. Marletta et al. also found that over 70% of students exceeded 4 h/day, a pattern linked to poor attention and procrastination [46]. Osorio-Molina et al. noted in their meta-analysis that most studies identified ≥4 h/day as a threshold for problematic use [34]. Zhou et al. further summarized that 70% of included studies examined daily smartphone use as a predictive factor, often in association with psychological symptoms [36].

Demographic moderators were also identified. Several studies noted that female gender, younger age, earlier years of study, and high daily smartphone usage were consistently associated with elevated nomophobia risk [34,44]. These variables often moderated the strength or direction of associations between psychological factors and nomophobia. Review findings reinforced these patterns. Zhou et al., synthesizing 39 studies across 15 countries, identified FoMO, anxiety, depression, low self-regulation, and high usage duration as the most consistent predictors of nomophobia, with social media engagement and perceived stress frequently acting as mediators [36]. Ramjan et al. emphasized early-stage nursing students’ vulnerability to digital dependency and emotional distress [35]. Osorio-Molina et al. confirmed the importance of psychological discomfort, emotional dysregulation, and time management issues as key antecedents [34]. While the findings demonstrate conceptual consistency, most studies employed cross-sectional designs and self-reported measures, which limit causal inference. Additionally, few studies included longitudinal or objective behavioural data, which constrains the understanding of how predictors develop over time. In summary, nomophobia in nursing students appears to result from an interplay of emotional vulnerability (anxiety, stress, alienation), behavioural patterns (FoMO, passive social media use, extended daily smartphone use), and individual differences in coping capacity and resilience. Mediated and moderated relationships are beginning to be explored, and further longitudinal research is needed to establish causal pathways.

### 3.6. Instruments Used to Assess Nomophobia

Across the 19 primary studies included in the review, the Nomophobia Questionnaire (NMP-Q) was the most widely used instrument to assess nomophobia among nursing students. Developed by Yildirim and Correia, the NMP-Q is a 20-item self-report scale that measures four dimensions of nomophobia: not being able to communicate, losing connectedness, not being able to access information, and giving up convenience [4]. Each item is rated on a 7-point Likert scale (1 = strongly disagree to 7 = strongly agree), yielding a total score ranging from 20 to 140. Higher scores indicate higher levels of nomophobia.

The NMP-Q was used in 17 out of 19 studies, either in its original English version or in culturally adapted translations. Reported Cronbach’s alpha values across studies indicated excellent internal consistency, generally ranging from 0.88 to 0.95. For example, Jose et al. reported α = 0.91 [44]; Bilgiç reported α = 0.92 [39]; and Akbari et al. reported α = 0.90 for the Persian version [50]. In most studies, severity levels were categorized according to established cut-offs: scores between 21 and 59 indicate mild nomophobia, scores between 60 and 99 indicate moderate, and scores between 100 and 140 reflect severe nomophobia. This classification was consistently applied in studies such as Gaber-Hamzaa et al., Conte et al., and Janatolmakan et al. [11,40,43].

Adaptations and validations of the NMP-Q were explicitly discussed in several studies. For example, Akin and Durmaz used the validated Turkish version of the scale, reporting a Cronbach’s α of 0.94 [37]. Similarly, Janatolmakan et al. applied a previously validated Persian version with high internal consistency [11]. A few studies briefly noted the process of translation and back-translation or the use of previously validated language versions, though not all provided detailed psychometric data.

Two studies employed alternative or complementary tools. Tuna et al. [16] used the NMP-Q alongside the Fear of Missing Out Scale (FoMOS), and Akın and Durmaz [37] used the Toronto Alexithymia Scale (TAS-20) to explore concurrent psychological constructs. However, in both cases, the NMP-Q remained the primary diagnostic tool for nomophobia.

The three included reviews also confirmed the predominance of the NMP-Q in the literature. Zhou et al., in their scoping review of 39 studies, reported that the vast majority employed the NMP-Q, but noted variability in cut-off use and scoring interpretations across studies [36]. Osorio-Molina et al., through meta-analytic synthesis, highlighted the consistency in using the NMP-Q and its psychometric robustness across cultural contexts [34]. Ramjan et al. mentioned the NMP-Q as the most frequently adopted tool in the studies reviewed, but did not report additional psychometric details [35].

## 4. Discussion

This integrative review highlights the widespread prevalence of moderate to severe nomophobia among nursing students across diverse sociocultural contexts, alongside its consistent associations with psychological distress, impaired academic functioning, and emotional dysregulation. The findings also identify a set of behavioural and emotional predictors and moderators that contribute to the development and maintenance of nomophobia in this population. Psychological distress emerged as the most consistently associated domain across studies. Nomophobia was positively correlated with depression, anxiety, stress, and feelings of alienation [7,43,50]. Additional literature further reinforces this pattern: Bernabé-Mateo et al. found that higher levels of nomophobia among nursing students were significantly associated with increased anxiety and poorer sleep quality, highlighting the link between problematic smartphone use and psychological distress in this population [52]. These findings are further corroborated by Baby et al., who examined nomophobia among medical students and found a significant positive correlation with loneliness and a negative correlation with self-esteem [53]. More than 80% of the participants exhibited moderate to severe levels of nomophobia, with over one-third reporting low self-esteem, and the majority presenting moderate loneliness. Although the study targeted medical undergraduates rather than nursing students, the similarities in age, academic pressure, and smartphone reliance provide a valuable basis for comparison. Their results reinforce the role of nomophobia as both a symptom and amplifier of psychosocial distress, echoing the emotional dysregulation and feelings of isolation reported in several of the included studies [7,43,50]. Tastan et al. added that students with high levels of smartphone dependence reported impaired interpersonal functioning and difficulty engaging in clinical interactions, reinforcing the association between nomophobia and psychological isolation [51]. Emotional dysregulation was another key theme, with alexithymia emerging as both a correlate and possible precursor [37,54]. These results support the conceptualization of nomophobia as a maladaptive coping response to underlying emotional vulnerability. Behavioural predictors and mediators such as fear of missing out (FoMO), impulsivity, and stress were repeatedly associated with nomophobia. Tuna et al. (2023) and Kargın demonstrated that FoMO significantly predicted nomophobia [16,45], while Bajamal confirmed that FoMO, anxiety, and social media use were significant predictors [55]. Gutiérrez-Puertas et al. also suggested that depression may mediate the relationship between FoMO and problematic smartphone use [9]. This relationship appears particularly pronounced in distance education settings, where nomophobia has been shown to co-occur with netlessphobia and fear of missing out, reinforcing emotional dependence on constant digital connectivity among nursing students [42]. These findings may also be interpreted through broader theoretical perspectives on behavioural addiction and stress–coping processes. In particular, the Interaction of Person–Affect–Cognition–Execution (I-PACE) model proposes that individual vulnerabilities, affective responses, and cognitive biases interact to reinforce problematic technology use behaviours [56]. Within this framework, factors such as fear of missing out, emotional dysregulation, and perceived stress may contribute to excessive smartphone reliance as a coping strategy, which may in turn interfere with academic engagement and professional learning. Furthermore, impulsivity, examined by Marletta et al., has been identified as a behavioural vulnerability that may predispose individuals to digital dependency [46].

These results align with those of Durak et al., who, in a large-scale study among adolescents, identified academic achievement, patterns of ICT use, and parental factors as significant correlates and statistical predictors of both nomophobia and smartphone addiction [57]. Although the population differs, these findings support the broader application of social-cognitive models and emphasize the need to consider both individual and environmental variables in understanding digital dependence. Some studies in other populations, such as medical or high-school students, have reported similar associations between smartphone overuse, anxiety, and reduced academic performance, suggesting a generalized pattern of maladaptive coping. While comparative evidence from non-nursing populations (e.g., medical students or adolescents) helps to support theoretical triangulation, population differences may limit the direct transferability of such findings to nursing students. Taken together, these findings support the interpretation of nomophobia as a multifactorial phenomenon shaped by interacting emotional, cognitive, and behavioural mechanisms. The impact of nomophobia on academic performance, attention, and decision-making was clearly documented. Students with high nomophobia reported increased procrastination, mental fatigue, and impaired concentration [40,44]. These findings are also consistent with previous evidence emphasizing the cognitive burden and academic disorganization associated with excessive smartphone use [58].

In clinical contexts, nomophobia was shown to impair decision-making and communication, especially among students with co-occurring alexithymia [37]. Sleep disturbances emerged as an important but underexplored consequence of excessive smartphone use. Studies by Haddaouy et al. and additional work on sleep disruption revealed that poor sleep quality and insomnia were significantly associated with smartphone overuse and emotional distress [59]. These psychophysiological consequences likely contribute to decreased academic and clinical performance, suggesting a need to address sleep hygiene in interventions targeting nomophobia. The validity of the diagnostic tools used to measure nomophobia also warrants discussion. All primary studies relied on self-report instruments, most commonly the NMP-Q. A recent cross-cultural psychometric study confirmed the internal consistency and four-factor structure of the NMP-Q across diverse populations [59]. However, the authors reported only partial measurement invariance across countries, raising concerns about the tool’s cultural adaptability and comparability. These concerns are echoed in individual studies that employed varying cut-off thresholds to define nomophobia severity [40,43]. Efstathiou et al. noted the lack of integration of objective behavioural measures or clinical interviews in the assessment of nomophobia, a limitation that affects the interpretability of current findings [60]. Finally, several theoretical and educational implications emerge. These findings resonate with broader conceptual frameworks of digital dependency. For instance, the model proposed by Kircaburun et al., though focused on problematic social media use, integrates personality traits, motivational drivers (e.g., emotional escape, social reward), and platform-specific use patterns, offering a useful lens through which to interpret behavioural predictors of nomophobia [61]. As emphasized by Ramjan et al. and Osorio-Molina et al., early interventions targeting digital dependency in nursing students are essential to preserve both mental well-being and clinical competence [34,35]. Overall, the evidence supports the interpretation of nomophobia as a multifactorial phenomenon with important psychological, academic, and professional implications for nursing students. While existing studies provide important insights, the predominance of cross-sectional designs and reliance on self-report tools limit causal inferences. Future research should prioritize longitudinal and interventional studies, incorporate objective behavioural assessments, and explore cross-cultural validation of diagnostic tools to deepen understanding and inform evidence-based interventions.

### 4.1. Psychophysiological Consequences: The Role of Sleep

An emerging but underexplored consequence of nomophobia among nursing students is its negative impact on sleep quality and psychophysiological functioning. While only a limited number of studies directly assessed sleep parameters using validated instruments, several authors noted indirect signs of sleep disruption. For instance, Bilgiç et al. and Jose et al. reported that students with higher levels of smartphone dependence experienced increased mental fatigue, academic procrastination, and concentration difficulties—symptoms often associated with poor sleep hygiene [39,44]. Similarly, Conte et al. described difficulty maintaining attention and alertness during academic tasks, particularly during examination periods, which may plausibly relate to reduced sleep quality [40]. These findings are reinforced and clarified by evidence from additional literature. Haddaouy et al. reported that excessive smartphone use—particularly in the pre-sleep period—was significantly associated with insomnia symptoms, delayed sleep onset, and reduced sleep duration [59]. Alzaher et al. similarly found that bedtime phone use disrupted circadian rhythms and worsened sleep quality, contributing to cumulative fatigue and mood instability [62]. In this regard, Moreno-Guerrero et al. emphasized that nomophobia is associated with reduced time allocated to rest and recovery, which may further exacerbate academic disorganization and cognitive overload among nursing students [48]. Consistent with these findings, Elbilgahy et al. reported that excessive use of electronic devices among nursing students was associated with poorer sleep quality and lower academic performance, highlighting the downstream cognitive and functional consequences of disrupted rest [41]. The systematic review by Mudgal et al. further contextualized these patterns, identifying sleep impairment as a secondary but clinically relevant outcome of nomophobia among healthcare students, albeit underreported in the existing research [63]. Collectively, these findings suggest that psychophysiological dysregulation, especially sleep disturbances, may act as a hidden but important mediator linking nomophobia with academic inefficiency and emotional dysregulation. Although not systematically captured by the included studies, this dimension appears crucial in understanding the full burden of smartphone dependence in nursing education. Future research should aim to integrate validated sleep assessment tools and explore causal pathways using longitudinal or experimental designs. Educational interventions may also benefit from incorporating modules on sleep hygiene and behavioural strategies to limit nighttime smartphone use, particularly in high-risk populations such as nursing students.

### 4.2. Professional Readiness and Clinical Competence

One of the most concerning findings to emerge from this review is the potential impact of nomophobia on clinical decision-making and the broader development of professional competence in nursing students. Several included studies suggested that excessive smartphone use and psychological dependence may interfere with cognitive control and judgement in clinical contexts. For example, Akın and Durmaz found that nursing interns with high levels of nomophobia and alexithymia reported greater difficulty in interpreting patient cues and prioritizing actions under pressure [37].

This aligns with findings by Conte et al., who noted a deterioration in task planning and organization among students with high nomophobia, especially during high-stress academic periods [40]. In line with these findings, Márquez-Hernández et al. reported that problematic mobile phone use and higher nomophobia levels were significantly associated with impaired clinical decision-making among nursing students, suggesting that digital dependence may interfere with cognitive processing and judgement during learning and care-related activities [47]. Similarly, Tangmunkongvorakul et al. [64] observed that increased smartphone addiction was associated with impaired decision-making and lower clinical performance among nursing students, further reinforcing concerns about the cognitive consequences of digital overuse in professional training [64]. Supplementary literature provides further insight and triangulation. The study by Demiralp et al. showed that problematic smartphone use was associated with decreased goal setting and reduced daily self-organization skills among nursing students [65]. These executive functioning impairments may translate into difficulties in managing clinical responsibilities and academic planning. Complementarily, Savcı et al. provided direct empirical evidence that smartphone addiction was negatively correlated with clinical decision-making skills (r = −0.16, *p* < 0.01). Their multivariate analysis revealed that both smartphone addiction and cyberloafing significantly predicted reduced decision-making ability, reinforcing the concern that digital dependency undermines core professional competencies [66]. These findings echo concerns expressed in the review by Zhou et al., which identified weakened clinical judgement and communication as potential long-term consequences of persistent smartphone dependence [36]. Additionally, the work of Tastan et al., included in the present review, emphasized the interpersonal ramifications of nomophobia [51]. Nursing students with high smartphone dependency reported greater discomfort in face-to-face interactions and reduced confidence in clinical communication—skills that are fundamental to safe and compassionate care. These findings were reinforced by the observational insights from Ramjan et al., who highlighted a worrying trend: students increasingly favour digital contact over in-person communication, a behaviour that may erode essential professional skills [35]. These concerns are echoed in the findings of Aguilera-Manrique et al. (2018), who reported a significant positive association between nomophobia levels and smartphone-related distractions among nursing students during their clinical practicum [67]. Notably, students with higher nomophobia scores were more likely to use their phones during patient care, despite acknowledging the necessity of restriction policies. This duality reflects the ambivalence found in other studies and underlines the clinical relevance of nomophobia as a potential barrier to professional behaviour and attentional control in healthcare environments. Collectively, these data support the hypothesis that nomophobia may negatively affect not only individual well-being and academic performance but also readiness for clinical practice. The combination of impaired concentration, emotional avoidance (alexithymia), and reduced interpersonal engagement could pose potential implications for patient safety and the therapeutic relationship. Educational programmes may address this risk through structured digital health literacy training, simulation-based decision-making exercises, and modules on self-awareness and emotional intelligence. Embedding such content early in nursing curricula may help to mitigate the risk of digital overreliance and promote the development of resilient, attentive, and ethically grounded professionals.

### 4.3. Nomophobia and Academic Burnout

Although burnout was not directly assessed in the studies included in this review, the psychological profile associated with nomophobia, characterized by persistent anxiety, emotional dysregulation, and cognitive overload, may represent a potential vulnerability pathway for academic exhaustion among nursing students. Evidence from related literature suggests that technology-related behaviours linked to fear of missing out and problematic smartphone use are associated with increased psychological strain and learning burnout in university students [15]. In addition, excessive smartphone use has been associated with poorer psychological well-being and reduced emotional balance among university populations [64]. Considering that nursing students are already exposed to substantial academic and emotional demands during their training, digital overreliance may further exacerbate stress-related responses and potentially contribute to early manifestations of academic burnout. Recent umbrella evidence on mental health in nursing students also highlights the high prevalence of psychological distress within this population, suggesting that additional stressors, such as maladaptive technology use, may represent an important but still underexplored factor [60]. Future research should therefore investigate the potential relationship between nomophobia, emotional exhaustion, and burnout using longitudinal designs.

### 4.4. Methodological Limitations of the Evidence

Several methodological limitations within the existing literature should be considered when interpreting these findings. Most of the included studies adopted cross-sectional designs, which limits the possibility of establishing causal relationships between nomophobia and psychological or academic outcomes. In addition, the majority of studies relied on self-reported measures of smartphone use and psychological variables, potentially introducing recall bias and social desirability bias. Many investigations were conducted within single institutions using convenience sampling strategies, which may reduce the generalisability of findings across different educational and cultural contexts. Furthermore, variability in measurement instruments, particularly between tools assessing nomophobia specifically (e.g., NMP-Q) and broader constructs of problematic smartphone use, may contribute to heterogeneity in prevalence estimates and associated outcomes. These methodological constraints highlight the need for future longitudinal and multicentre studies employing harmonized assessment tools and objective behavioural indicators.

### 4.5. Educational Implications and Interventions

The evidence highlights the urgent need to integrate structured interventions into nursing curricula across three complementary domains:

Critical Digital Literacy: Nomophobia has been linked to impaired attention, reduced motivation, and lower academic performance among nursing students [17,36,40,43]. Educational programmes should promote functional and mindful use of mobile technologies, raising awareness of the risks associated with hyperconnectivity and cognitive distraction [68].

Training in Emotional Regulation: Difficulties in emotion regulation, anxiety, and depression are consistently associated with nomophobia [7,37,54]. Interventions focused on emotional intelligence and adaptive coping are essential to mitigate these risks [69,70,71].

Clinical simulations with digital stressors: Studies show that nomophobia negatively affects concentration and clinical decision-making in healthcare contexts [37,40], as well as in supplementary contributions [65,66], suggesting the usefulness of simulation scenarios in which cell phone use is included as a disruptive factor. Such exercises can help assess and improve decision-making readiness in realistic clinical contexts [72].

Potential directions for intervention: Evidence from related fields suggests that digital well-being programmes, psychoeducational approaches, and self-regulation strategies may represent promising directions for addressing problematic smartphone use among university students. However, intervention studies specifically targeting nomophobia in nursing students remain scarce, and the effectiveness of such approaches in this population has yet to be established [73,74].

Finally, institutional policies that promote focused learning environments and encourage responsible technology use during both academic and clinical training may help mitigate digital dependency, strengthen professional readiness, and support safe and effective nursing practice. Taken together, these findings position nomophobia not merely as a behavioural phenomenon related to smartphone use, but as a multidimensional issue intersecting psychological well-being, academic functioning, and professional development within nursing education.

#### Directions for Future Research

Despite offering an integrated overview, this review reveals important methodological gaps in the current literature:

- Longitudinal and Experimental Designs: Most available studies adopt cross-sectional approaches, limiting causal inference. Longitudinal and interventional research is needed to clarify trajectories and evaluate preventive programmes [34,35].
- Objective Measures of Digital Behaviour: Nomophobia research relies heavily on self-report questionnaires. Integrating digital tracking, ecological momentary assessments, or observational tools is recommended to improve validity [60].
- Environmental and Cultural Factors: Cultural and family contexts significantly influence nomophobia expression, underscoring the need for culturally sensitive [43,57,68,75].
- Cross-cultural Validation of Diagnostic Tools remains a critical issue: although the Nomophobia Questionnaire (NMP-Q) is widely used, concerns persist regarding cultural adaptation, measurement invariance, and variability in cut-off scores across different populations [76].
- Extension to Other Health Professions: Recent findings confirm that digital dependency and its psychological risks are shared across medical, nursing, and allied health students [49]. Expanding research beyond nursing to include medical, midwifery, and physiotherapy students would enable the identification of common patterns and the development of systemic, interprofessional preventive strategies.

## 5. Interpretative Synthesis and Conceptual Framework

Building upon the convergent evidence synthesized in this review, the findings were integrated into a conceptual framework informed by the Interaction of Person–Affect–Cognition–Execution (I-PACE) model of behavioural addictions proposed by Brand et al. [56]. The framework also draws on principles from stress–coping theory [77], acknowledging that nomophobia may emerge as a maladaptive emotion-focused coping response to academic and clinical stressors experienced by nursing students. According to this conceptual interpretation, individual vulnerabilities—such as emotional dysregulation, psychological distress, and alexithymia—may interact with contextual pressures related to academic workload, clinical training demands, and constant digital connectivity. These factors may influence affective and cognitive mediators, including fear of missing out (FoMO), impulsivity, anxiety, and sleep disturbance, which in turn may contribute to excessive smartphone reliance and the development of nomophobia. Within this framework, nomophobia represents a central phenomenon arising from the interaction between individual predispositions and maladaptive coping processes. The literature synthesized in this review further suggests that nomophobia may be associated with a range of academic, psychological, and professional outcomes among nursing students, including reduced concentration, emotional distress, sleep disturbance, and potential challenges in clinical decision-making and interpersonal communication. Protective factors such as resilience, self-regulation, and digital health literacy may act as buffering mechanisms that mitigate the impact of these pathways. The proposed framework, therefore, provides an interpretative synthesis linking individual vulnerabilities, behavioural mediators, and educational outcomes associated with nomophobia in nursing students.

**Figure 2:**
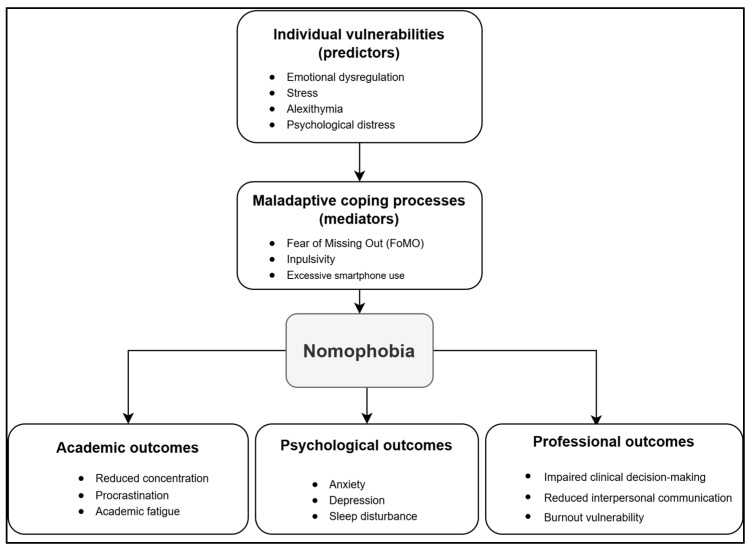
Conceptual framework of predictors, mediators, and outcomes of nomophobia in nursing students (informed by the I-PACE model).

Figure 2 summarizes this conceptual framework, illustrating the potential pathways connecting predictors, mediators, and outcomes identified across the included studies. The framework should be interpreted as a conceptual synthesis derived from the literature rather than a causal model, and it highlights directions for future longitudinal and interventional research. By organizing the available evidence into an integrated conceptual structure, this framework not only synthesizes the relationships identified in the literature but also generates theoretically grounded hypotheses that may guide future empirical investigations on nomophobia among nursing students.

## 6. Conclusions

This integrative review demonstrates that nomophobia is a highly prevalent condition among nursing students, with moderate-to-severe levels reported in up to 90% of participants across diverse cultural contexts. Evidence consistently shows that nomophobia is associated with psychological distress—most notably anxiety, depression, stress, insomnia, fear of missing out (FoMO), and alexithymia—as well as reduced resilience and feelings of social alienation. Excessive smartphone use is further linked to impaired academic performance, decreased attention and concentration, procrastination, and difficulties in clinical decision-making, with potential implications for patient safety and professional competence. Behavioural and psychological predictors such as FoMO, perceived stress, extended daily smartphone use, and younger age or female gender appear to play a central role in shaping vulnerability. The convergence of findings across the available evidence highlights the urgent need for systemic educational strategies that integrate digital literacy, emotional regulation training, resilience-building, and high-fidelity simulation. Future research should employ longitudinal and interventional designs to establish causal pathways and evaluate the effectiveness of tailored interventions. Therefore, addressing nomophobia is essential not only for safeguarding students’ mental health and academic success but also for supporting the development of clinical reasoning, professional readiness, and patient safety competencies in the future nursing workforce.

## 7. Limitations

This integrative review is subject to several limitations that warrant careful consideration. First, most included studies adopted cross-sectional designs, which restrict the capacity to infer causal relationships between nomophobia and its psychological, behavioural, and academic correlates. Longitudinal and interventional designs remain scarce, limiting understanding of developmental trajectories and the effectiveness of preventive interventions. Moreover, most studies relied on non-probabilistic sampling, often from single institutions, raising concerns about selection bias and generalizability. Second, there are significant methodological issues related to measurement. The widespread reliance on self-report instruments, predominantly the Nomophobia Questionnaire (NMP-Q), introduces the risk of response and social desirability biases, particularly in culturally sensitive domains such as technology-related dependence. While the NMP-Q demonstrates strong internal consistency (α = 0.88–0.95 across studies), only a subset of investigations reported formal validation or cultural adaptation processes, undermining comparability across settings. Furthermore, inconsistent application of severity cut-offs contributes to variability in prevalence estimates. Third, the near-total absence of objective behavioural indicators (e.g., digital tracking, ecological momentary assessments) or clinical diagnostic interviews restricts the capacity to triangulate self-reported nomophobia with real-world behaviours or clinically significant impairment. This gap limits the interpretability of current findings in terms of clinical relevance and practical implications. Finally, potential moderators and confounders, including age, gender, socioeconomic status, academic stress, and pre-existing mental health conditions, were inconsistently analyzed or statistically controlled. This omission weakens the robustness of multivariate models and obscures nuanced subgroup patterns.

## Figures and Tables

**Table 1:** Summary of the included studies on nomophobia and related problematic smartphone-use constructs.

Study	Country	Design	Sample	Main Evidence
Osorio-Molina et al. (2021) [34]	International	Systematic review and meta-analysis	16 studies; 8 in meta-analysis (pooled *n* = 2780)	Smartphone addiction was common among nursing students; pooled prevalence was 39.7%, with associations with female gender, anxiety, poor sleep, and worse academic outcomes.
Ramjan et al. (2021) [35]	International	Integrative review	27 studies	Consistent evidence showed problematic smartphone use/nomophobia, distraction in classroom and clinical settings, poorer sleep, and links with stress, anxiety, depression, loneliness, and reduced communication skills.
Zhou et al. (2024) [36]	International	Scoping review	39 studies across 15 countries	Prevalence ranged from 19% to 72%; harms included poorer sleep, anxiety/depression, lower self-esteem, poorer learning/attention, and interpersonal difficulties; risk factors aligned with I-PACE domains.
Akın and Durmaz (2024) [37]	Turkey	Cross-sectional correlational	*n* = 207 nursing students	Moderate/extreme nomophobia was common and was positively associated with alexithymia, especially difficulty identifying and expressing emotions.
Anand et al. (2022) [38]	India	Descriptive cross-sectional	*n* = 643 nursing students	Most students had mild to moderate nomophobia; severity was associated with year of study, daily smartphone use, and phone-checking frequency.
Berdida and Grande (2023) [17]	Philippines	Cross-sectional with SEM	*n* = 835 nursing students (SEM analysis on *n* = 579)	Nomophobia had negative effects on attention and motivation, and indirectly affected academic performance through reduced attention.
Bilgiç et al. (2024) [39]	Turkey	Descriptive cross-sectional	*n* = 541 nursing students	Higher smartphone addiction was associated with more frequent use/checking, lower peer relationship scores, and more health complaints, such as headache and sleep problems.
Çobanoğlu et al. (2021) [6]	Turkey	Descriptive correlational cross-sectional	*n* = 215 nursing students	Nomophobia was moderately correlated with smartphone and digital addiction; these variables significantly predicted nomophobia and explained a substantial proportion of its variance.
Conte et al. (2023) [40]	Italy	Cross-sectional observational	*n* = 293 nursing students	Moderate/severe nomophobia was frequent and was positively associated with internet addiction, anxiety, and depression; higher use was observed in women and in those using phones >5 h/day.
Elbilgahy et al. (2021) [41]	Egypt/Saudi Arabia	Comparative cross-sectional	*n* = 920 female nursing students (Egypt and Saudi Arabia)	Greater internet/electronic device addiction was associated with excessive daytime sleepiness and poorer academic performance, especially in the Saudi sample.
Eskin Bacaksız et al. (2022) [42]	Turkey	Descriptive cross-sectional	*n* = 802 nursing students	Nomophobia was positively associated with netlessphobia and FoMO; both variables predicted nomophobia during distance education.
Gaber-Hamzaa et al. (2024) [43]	Egypt	Descriptive cross-sectional multisite survey	*n* = 1273 nursing students	Moderate and severe nomophobia were highly prevalent; nomophobia was positively associated with psychological alienation and negatively associated with academic performance.
Jose et al. (2024) [44]	India	Descriptive cross-sectional	*n* = 402 nursing students	Problematic mobile phone use was positively associated with depression and insomnia, and negatively associated with self-esteem and life satisfaction.
Kargın et al. (2020) [45]	Turkey	Descriptive cross-sectional	*n* = 511 nursing students	Internet addiction and fear of missing out were positively correlated, suggesting a potential vulnerability pathway for problematic digital behaviours among nursing students.
Marletta et al. (2021) [46]	Italy	Cross-sectional observational	*n* = 244 nursing students (Italy)	Higher daily smartphone use was associated with higher nomophobia scores; nomophobia was common across years of study.
Márquez-Hernández et al. (2020) [47]	Spain	Cross-sectional correlational	*n* = 124 nursing students	Nomophobia and problematic phone use were strongly related; higher nomophobia was associated with lower decision-making confidence and more avoidant/procrastinating decision styles.
Moreno-Guerrero et al. (2021) [48]	Spain	Descriptive, correlational, predictive, cross-sectional	*n* = 596 nursing students	Nomophobia was linked to poorer rest/sleep-related variables; greater smartphone use predicted a more negative impact on sleep.
Sadeghi et al. (2025) [18]	Iran	Descriptive-analytical cross-sectional	*n* = 258 nursing students	All students reported at least mild nomophobia, and nomophobia was positively associated with social interaction anxiety.
Tárrega-Piquer et al. (2023) [49]	Spain	Cross-sectional observational	*n* = 304 nursing students	Moderate/severe nomophobia was common and was positively associated with social anxiety and academic procrastination; higher scores were found in students using phones >5 h/day.
Tuna et al. (2023) [16]	Turkey	Analytical cross-sectional	*n* = 802 nursing students	Netlessphobia and FoMO explained a substantial proportion of nomophobia variance; smartphone/internet use was high during distance education.
Yigit et al. (2024) [7]	Turkey	Cross-sectional correlational	*n* = 544 nursing students	Higher nomophobia was associated with greater depression, anxiety, and stress; higher scores were also linked with specific smartphone-related habits.
Janatolmakan et al. (2024) [11]	Iran	Cross-sectional correlational	*n* = 500 nursing students	Nomophobia was frequent (81.4%); non-academic smartphone use was associated with higher nomophobia, and smartphone-use patterns were described in relation to academic performance.

## Data Availability

No new data were created or analyzed in this study. Data sharing is not applicable.

## Supplementary Materials

The following supporting information can be downloaded at: https://www.mdpi.com/article/10.3390/healthcare14070830/s1, Table S1: Complete search strategies for all databases; Table S2a: Quality appraisal of included reviews using the QuADS tool; Table S2b: Quality appraisal of cross-sectional studies using the JBI checklist; Table S3: Data extraction of included review studies; Table S4: Data extraction of included quantitative studies.

## Abbreviations

The following abbreviations are used in this manuscript:

FoMo	Fear of Missing Out
JBI	Joanna Briggs Institute
NMP-Q	Nomophobia Questionnaire
PRISMA	Preferred Reporting Items for Systematic Reviews and Meta-Analyses
QuADS	Quality Assessment with Diverse Studies
