# Supplementary material for: Nomophobia in Nursing Students: Psychological, Academic, and Clinical Impacts—An Integrative Review
Source: Healthcare (Basel). 2026 Mar 24;14(7):830. doi: 10.3390/healthcare14070830 (PMC13072822; doi:10.3390/healthcare14070830)
Supplement: Supplementary file 1 [file healthcare-14-00830-s001.zip › healthcare-4148443-supplementary.pdf]

Table S1. Search Strategy

Database	Search String	Search results
**PubMed**	(("Nomophobia"[Title/Abstract] OR "smartphone addiction"[Title/Abstract] OR "problematic smartphone use"[Title/Abstract] OR "mobile phone dependence"[Title/Abstract]) AND ("students, nursing"[MeSH Terms] OR "nursing students"[Title/Abstract] OR "student nurses"[Title/Abstract])) AND (y_10[Filter])	69
	(("Nomophobia"[All Fields] OR "smartphone addiction"[All Fields] OR "problematic smartphone use"[All Fields] OR "mobile phone overuse"[All Fields]) AND ("nursing student*"[All Fields] OR "student nurse*"[All Fields] OR "undergraduate nursing"[All Fields]) AND ("health care"[All Fields] OR "healthcare"[All Fields] OR "mental health"[All Fields] OR "well-being"[All Fields])) AND (y_10[Filter])	28
	(("Nomophobia"[Title/Abstract] OR "smartphone addiction"[Title/Abstract] OR "problematic smartphone use"[Title/Abstract] OR "mobile phone dependence"[Title/Abstract]) AND ("students, nursing"[MeSH Terms] OR "nursing students"[Title/Abstract] OR "student nurses"[Title/Abstract]) AND ("academic performance"[Title/Abstract] OR "academic achievement"[Title/Abstract] OR "learning outcomes"[Title/Abstract] OR "student performance"[Title/Abstract] OR "decision-making"[Title/Abstract] OR "clinical skills"[Title/Abstract] OR "attention"[Title/Abstract] OR "concentration"[Title/Abstract]) AND ("academic performance"[Title/Abstract] OR "academic achievement"[Title/Abstract] OR "learning outcomes"[Title/Abstract] OR "student performance"[Title/Abstract] OR "decision-making"[Title/Abstract] OR "clinical skills"[Title/Abstract] OR "attention"[Title/Abstract] OR "concentration"[Title/Abstract] OR "educational outcomes"[Title/Abstract] OR "cognitive performance"[Title/Abstract])) AND (y_10[Filter])	20
**Medline**	(nomophobia or problematic mobile phone use or mobile phone addiction) AND (student nurses or nursing students or student nurse or nursing student or undergraduate nurse) AND (academic achievement or academic performance or academic success)	89
**CINAHL**	nomophobia OR (problematic mobile phone use or mobile phone addiction) AND (student nurses or nursing students or student nurse or nursing student or undergraduate nurse) AND (mental health or mental illness or mental disorder or psychiatric illness or anxiety or depression or well-being or distress)	145
**PsycInfo**	(nomophobia or problematic mobile phone use or mobile phone addiction) AND (student nurses or nursing students or student nurse or nursing student or undergraduate nurse) AND (academic achievement or academic performance or academic success)	2
	(nomophobia or problematic mobile phone use or mobile phone addiction) AND (student nurses or nursing students or student nurse or nursing student or undergraduate nurse) AND (mental health or mental illness or mental disorder or psychiatric illness or anxiety or depression or well-being or distress)	8

Table S2a: QuADS Quality Appraisal for Diverse Studies* (adapted from [33])

**QuADS**<br>***Quality Appraisal for diverse studies***	**Osorio-Molina et al. (2021) [34]**	**Ramjan et al. (2021) [35]**	**Zhou et al. (2024) [36]**
**1** Theoretical or conceptual underpinning to the research	2	3	3
**2** Statement of research aim/s	3	3	3
**3** Clear descriptions of research setting and target population	2	3	3
**4** The study design is appropriate to address the stated research aim/s	3	3	3
**5** Appropriate sampling to address the research aim/s	2	3	3
**6** Rationale for choice of data collection tool/s	2	2	2
**7** The format and content of data collection tool is appropriate to address the stated research aim/s	2	2	2
**8** Description of data collection procedure	2	2	2
**9** Recruitment data provided	2	3	2
**10** Justification for analytic method selected	2	2	2
**11** The method of analysis was appropriate to answer the research aim/s	2	2	2
**12** Evidence that the research stakeholders have been considered in research design or conduct.	0	0	0
**13** Strengths and limitations critically discussed	3	3	2
**Quality scores**	**25/39**<br>**Quality=Moderate**	**31/39**<br>**Quality=High**	**29/39**<br>**Quality=Moderate**

*Rationale for using QuADS on reviews: QuADS items were adapted to the review context: sampling ↦ search/selection strategy; data collection ↦ data extraction/charting; analysis ↦ narrative synthesis / meta-analysis. This adaptation was pre-specified to ensure consistent appraisal across heterogeneous designs

Table S2b Quality appraisal of cross-sectional studies using the JBI Critical Appraisal Checklist.

**Study**	**Were the criteria for inclusion in the sample clearly defined?**	**Were the study subjects and the setting described in detail?**	**Was the exposure measured in a valid and reliable way?**	**Were objective, standard criteria used for measurement of the condition?**	**Were confounding factors identified?**	**Were strategies to deal with confounding factors stated?**	**Were the outcomes measured in a valid and reliable way?**	**Was appropriate statistical analysis used?**	**Score**
**Janatolmakan et al., 2024 [11]**	Yes	Yes	Yes	Yes	Yes	Unclear	Yes	Yes	**7/8**
**Berdida and Grande (2023) [17]**	Yes	Yes	Yes	Yes	Unclear	No	Yes	Yes	**6/8**
**Eskin Bacaksiz et al. (2022) [42]**	Yes	Yes	Yes	Yes	No	No	Yes	Yes	**6/8**
**Tárrega-Piquer et al. (2023) [49]**	Yes	Yes	Yes	Yes	No	No	Yes	Yes	**6/8**
**Jose et al. (2024) [44]**	Yes	Yes	Yes	Yes	No	No	Yes	Yes	**6/8**
**Tuna et al. (2023) [16]**	Yes	Yes	Yes	Yes	No	No	Yes	Yes	**6/8**
**Anand et al. (2022) [38]**	Yes	Yes	Yes	Yes	No	No	Yes	Yes	**6/8**
**Sadeghi et al. (2025) [18]**	Yes	Yes	Yes	Yes	No	No	Yes	Yes	**6/8**
**Márquez-Hernández et al. (2020) [47]**	Yes	Yes	Yes	Yes	No	No	Yes	Yes	**6/8**
**Bilgiç et al. (2024) [39]**	Yes	Yes	Yes	Yes	No	No	Yes	Yes	**6/8**
**Çobanoğlu et al. (2021) [6]**	Yes	Yes	Yes	Yes	No	No	Yes	Yes	**6/8**
**Conte et al. (2023) [40]**	Yes	Yes	Yes	Yes	No	No	Yes	Yes	**6/8**

**Moreno-Guerrero et al. (2021) [48]**	Yes	Yes	Yes	Yes	No	No	Yes	Yes	**6/8**
**Elbilgahy et al. (2021) [41]**	Yes	Yes	Yes	Yes	No	No	Yes	Yes	**6/8**
**Gaber-Hamza et al. (2024) [43]**	Yes	Yes	Yes	Yes	No	No	Yes	Yes	**6/8**
**Kargın et al. (2020) [45]**	Yes	Yes	Yes	Yes	No	No	Yes	Yes	**6/8**
**Marletta et al. (2021) [46]**	Yes	Yes	Yes	Yes	No	No	Yes	Yes	**6/8**
**Yigit et al. (2024) [7]**	Yes	Yes	Yes	Yes	No	No	Yes	Yes	**6/8**
**Akin et al. (2024) [37]**	Yes	Yes	Yes	Yes	No	No	Yes	Yes	**6/8**

Table S3. Data extraction table for included review studies

N°	Authors (Year of publication)	Country	Title	Study Type and Aim	Context and Sample	Data Collection Tool	Main Results	Declared Limitations	QuADS
1	**Osorio-Molina et al. (2021)** [34]	International	Smartphone addiction, risk factors and its adverse effects in nursing students: A systematic review and meta-analysis	Systematic Review and Meta-analysis<br><br>**Aim:** to estimate the pooled prevalence of smartphone addiction among nursing students and to identify associated risk factors and adverse effects.	16 studies (qualitative synthesis); 8 studies in the meta-analysis (pooled n = 2,780). Studies were conducted mainly in Türkiye, South Korea, Spain, and the United States, with additional samples from Portugal, Iran, China, and the UK	Standardized instruments (e.g., SAS, SAS-SV), pooled through meta-analytic techniques.	– Pooled prevalence of smartphone addiction: 39.7% (95% CI 30.3–49.6).<br>– Higher prevalence in Asia than in Europe.<br>– Significant associations: female gender, anxiety, poor sleep quality, academic underperformance.<br>– Smartphone addiction linked to negative academic and mental-health outcomes.	High heterogeneity across studies; possible publication bias; varied assessment tools; predominantly cross-sectional designs.	Quality=Moderate<br><br>25/39
2	**Ramjan et al. (2021) [35]**	International	The negative impact of smartphone usage on nursing students: An integrative literature review	Integrative review<br><br>**Aim:** to synthesise the negative effects of smartphone use/nomophobia on nursing students across classroom and clinical settings	27 studies; university and clinical settings across multiple countries (Korea, Türkiye, India, Spain, USA, etc.). Total participants ≈10,200 (range 8–1,446 per study; mean 378±331; estimated from per-study Ns).	Scales for nomophobia/PSU (NMP-Q; SAS/SAS-SV; MPPUS/PMPU), sleep (PSQI), distress (DASS/HADS), attention/learning measures, and ad-hoc questionnaires on usage hours/distraction	Descriptive synthesis (no pooled estimates). Nomophobia/PSU common; examples from included studies: moderate 51.9%, severe 13.6% (NMP-Q, large sample). Higher use/PSU ⇒ poorer sleep quality (e.g., r ≈ .30; β = .152; p < .001).<br><br>-Distraction in classroom/clinical placement; in a quasi-experiment, removing	Predominantly cross-sectional designs; heterogeneity of tools/cut-offs; self-report measures; exclusion of non-English and grey literature.	Quality=High<br><br>31/39

							smartphones ⇒ increased attention and test scores.<br><br>-Associations with stress/anxiety/depression and loneliness/social distress; inverse associations with social support, life satisfaction, self-esteem, and communication skills.		
**3**	**Zhou et al. (2024) [36]**	International	A model for risk factors and harms of smartphone addiction among nursing students: a scoping review.	Scoping review<br><br>**Aim:** to map prevalence, harms (physiological/psychological/social), and risk factors of smartphone addiction among nursing students, integrating Engel and I-PACE frameworks.	39 studies across 15 countries (searches up to August 2023); total participants not specified (per-study Ns listed in the review).	Standard addiction measures (e.g., SAS/SAS-SV, PMPU/MPPUS); outcome measures: sleep, anxiety/depression, self-esteem/learning/attention, interpersonal relations; thematic synthesis with no formal quality appraisal	-Descriptive evidence only (no meta-analysis): prevalence 19–72% (typically 40–50%); qualitative links with poorer sleep, more anxiety/depression, lower self-esteem/learning/attention;<br><br>risk factors per I-PACE (stress/burnout, low social support, poor self-control, prolonged/bedtime use). High heterogeneity; mostly cross-sectional studies.	Heterogeneous and partly descriptive evidence; some small samples; no formal quality appraisal (by design for scoping reviews).	Quality=Moderate<br><br>29/39

Table S4. Data extraction table for included quantitative studies

N°	Authors (Year of publication)	Country	Title	Study Type and Aim	Sample and setting	Data collection tool	Main Results	Declared Limitations	JBI
1	**Akın et al. (2024) [37]**	Turkey	The relationship between nomophobia and alexithymia levels in nurse interns: descriptive study	Quantitative – Descriptive correlational cross-sectional study<br><br>**Aim:** to examine the relationship between nomophobia and alexithymia levels in undergraduate nursing students.	Country: Turkey<br>Sample size: 207 nursing students<br>Gender: 83.1% female<br>Age: Mean = 22.16, SD = 1.78<br>Sampling: Convenience sampling via Google Forms	Nomophobia Scale (NMP-Q, Turkish validated version)<br>Toronto Alexithymia Scale (TAS-20)	• Nomophobia mean score: 69.55 ± 27.74<br>-Moderate nomophobia: 44%<br>-Extreme nomophobia: 15%<br>• Alexithymia mean score: 51.12 ± 11.15<br>• Prevalence of alexithymia: 28%<br>• Correlation:<br>-Significant positive correlation between nomophobia and alexithymia ($p < .05$)<br>-Especially linked with difficulty identifying and expressing emotions (TAS-20 subdimensions)	Use of self-report measures (subject to bias)<br>Cross-sectional design (no causal inference)<br>Conducted in a single country; generalizability is limited	**Quality Rating Moderate**<br>→Strengths: Validated scales, clear objective, correlation analysis<br>→Limitations: no confounder adjustment, self-selection bias
2	**Anand et al. (2022) [38]**	India	A Study to Assess the Prevalence of Nomophobia among Nursing Students in Kollam	Descriptive cross-sectional study<br><br>**Aim:** to assess the prevalence and severity of nomophobia among nursing students and examine its associations with behavioral and demographic variables.	643 undergraduate nursing students from four private nursing colleges in Kollam, Kerala (India).<br>Sampling method: purposive (non-probability)	Nomophobia Questionnaire (NMP-Q), self-administered.<br><br>Statistical analysis: descriptive statistics and chi-square tests for associations ($p < 0.05$)	Nomophobia severity:<br>• Severe: 2%<br>• Moderate: 33%<br>• Mild: 54%<br>• No nomophobia: 11%<br>Overall prevalence (moderate + severe): 34.84%<br>Significant associations with:<br>• Year of study<br>• Hours of daily smartphone use<br>• Frequency and number of phone checking episodes per day<br>• Instrument reliability: Cronbach's $\alpha = 0.93$	-Non-random sampling<br>-Limited to a single geographic region (Kollam)<br>-Cross-sectional design prevents causal inference	**Quality Rating Moderate**<br>→ Strengths: large sample, validated tool, appropriate statistical analysis<br>→ Limitations: selection bias, limited generalizability, observational design

**3**	**Berdida and Grande (2023)**<br>**[17]**	Philippines	Nursing students' nomophobia, social media use, attention, motivation, and academic performance: A structural equation modeling approach	Cross-sectional correlational study using Structural Equation Modeling (SEM)<br><br>**Aim:** to assess the direct and indirect effects of nomophobia and social media use on attention, motivation, and academic performance among nursing students.	835 nursing students from three nursing colleges in the Philippines (full survey respondents)<br>579 students included in SEM analysis (complete and valid data)<br>Gender: 667 females (79.9%), 168 males (20.1%)<br>Mean age: 20.39 ± 1.63 years<br>All academic years represented (1st–4th year)<br>75.6% reported smartphone use >5 hours/day	Nomophobia Questionnaire (NMP-Q)<br>Social Media Engagement Questionnaire<br>Attention and Motivation Scales<br>Academic performance: self-reported GPA<br><br>Analysis: Confirmatory Factor Analysis (CFA), Structural Equation Modeling (SEM)	• Mean NMP-Q score: 76.09 ± 22.62 (moderate-to-high nomophobia)<br>• Nomophobia had a negative direct effect on attention ($\beta = -0.135$) and motivation ($\beta = -0.125$)<br>• No direct effect on GPA, but significant indirect effect via attention<br>• Social media use negatively affected attention ($\beta = -0.181$)<br>• Attention was the strongest predictor of GPA ($\beta = 0.250$)<br><br>SEM model showed excellent fit: $\chi^2/df = 1.56$, RMSEA = 0.05, CFI = 0.95	GPA was self-reported<br>Cross-sectional design (no causality)<br>Regional sample may limit generalizability	**Quality Rating**<br>**High**<br>→Strengths: large and diverse sample, advanced SEM analysis, validated instruments<br>→Limitations: self-reported GPA, cross-sectional design
**4**	**Bilgiç et al. (2024)**<br>**[39]**	Turkey	Smartphone Addiction and Peer Relations in Nursing Students	Descriptive, cross-sectional<br><br>**Aim:** to determine the levels of smartphone addiction among nursing students and to explore its effect on peer relationships.	Faculty of Health Sciences, Trakya University, Turkey<br>Total participants: 541 nursing students (response rate: 79.2%)<br>Mean age: 20.17 ± 1.75 years<br>78.9% female, 21.1% male	Smartphone Addiction Scale – Short Version (SAS-SV)<br>Peer Relationships Scale (PRS)	• Mean SAS-SV: 26.10 ± 11.16 (low).<br>• Mean PRS: 67.35 ± 12.43 (above average).<br>• Daily use & checking ↑ → SAS-SV ↑.<br>• Self-perceived "addicted": 34.01 vs 23.47 SAS-SV.<br>• SAS-SV ↔ PRS: $r=-0.170$ ($p<.001$).<br>• 36.2% reported health problems (e.g., headache, sleep), associated with higher SAS-SV.	Self-reported data<br>Single-center study<br>Small proportion of male participants	**Quality Rating**<br>**Moderate**<br>→Strengths: large sample, validated scales, detailed analysis<br>→Limitations: no control for confounders, single-site, self-reported data
**5**	**Çobanoğlu et al. (2021)**<br>**[6]**	Turkey	The relationship between nursing	Quantitative – Descriptive, correlational cross-sectional study	Country: Turkey<br>University: One public university	Nomophobia Questionnaire (NMP-Q)	Positive moderate correlations:<br>• NMP-Q with SAS-SV: $r = 0.628$	Single university sample limits generalizability	**Quality Rating**<br>**Moderate**<br>→Strengths: clear statistical

			students' digital and smartphone addiction levels and nomophobia: A descriptive, correlational study	**Aim:** to determine the relationship between nomophobia and perceived loneliness in nursing students.	in northeastern Turkey<br>Sample size: 215 nursing students<br>Age: Mean = 20.91 ± 2.06 (range 18–33)<br>Gender: 76.3% female<br>Year of study: 1st year = 32.6%, 2nd = 24.2%, 3rd = 20.9%, 4th = 22.3%	Smartphone Addiction Scale–Short Version (SAS-SV)<br>Digital Addiction Scale (DAS)	• NMP-Q with DAS: r = 0.518<br>Regression findings:<br>• SAS-SV (smartphone addiction) had a significant predictive effect on nomophobia (β = 0.765, p < .01)<br>• DAS (digital addiction) also significantly predicted nomophobia (β = 0.518, p < .01)<br>• Subdimensions of both scales contributed differently to nomophobia facets such as:<br>Inability to access information (β = 0.635)<br>Losing connectivity (β = 0.568)<br>Giving up convenience and not being able to communicate<br>Effect sizes:<br>• Smartphone addiction and digital addiction explained 47% of the variance in nomophobia<br>• Digital addiction alone explained 29%.	Self-reported measures may introduce bias<br>Cross-sectional design prevents causal inference	modeling, multiple validated tools, predictive analysis<br>→Limitations: convenience sample, self-report bias, no confounder control
**6**	**Conte et al. (2023) [40]**	Italy	Internet addiction, nomophobia, anxiety, and depression levels related: observational study among a population of Italian nursing students	Cross-sectional observational study<br><br>**Aim:** to investigate the levels of internet addiction, nomophobia, anxiety, and depression, and their interrelations in a sample of Italian nursing students.	293 nursing students from an Italian university<br>Mean age: 21.4 ± 3.1 years<br>83% female<br>Convenience sampling during academic year 2021–2022	Nomophobia Questionnaire (NMP-Q)<br>Internet Addiction Test (IAT)<br>Hospital Anxiety and Depression Scale (HADS)<br>Online questionnaire	Nomophobia:<br>• Mean score: 64.45 ± 21.79 (moderate)<br>• 61.8% moderate nomophobia<br>• 14.7% severe<br>Anxiety:<br>• 39.2% had borderline or abnormal anxiety (HADS-A ≥ 8)<br>Depression:	Self-reported data<br>Cross-sectional design<br>Single academic institution<br>Possible selection bias from convenience sampling	**Quality Rating Moderate**<br>→Strengths: strong psychometric tools, detailed correlations<br>→Limitations: no control for confounders, convenience

						Statistical analysis: Pearson correlation, t-test, ANOVA	• 28.3% showed depressive symptoms (HADS-D ≥ 8)<br>Internet Addiction:<br>• 47.4% had moderate addiction<br>Correlations:<br>• Strong positive correlation between nomophobia and internet addiction ($r = 0.632$, $p < .01$)<br>• Nomophobia also positively correlated with anxiety ($r = 0.579$) and depression ($r = 0.436$), all $p < .01$<br>Women and students using phones more than 5 hours/day had significantly higher nomophobia and IAT scores		sample, self-report
**7**	**Elbilgahy et al. (2021) [41]**	Egypt and Saudi Arabia	Effects of Electronic Devices and Internet Addiction on Sleep and Academic Performance Among Female Egyptian and Saudi Nursing Students: A Comparative Study	Comparative cross-sectional study<br><br>**Aim:** to investigate the prevalence of Internet and electronic device addiction among Egyptian and Saudi nursing students, with the goal of identifying any effects on sleep and academic performance.	In this study were recruited 920 female nursing students from Egypt and Saudi Arabia. Allowing for a 10% non-response rate, the estimated sample size was 264 ($240 + 240 \times 10\%$) for the Saudi students and 528 ($480 + 480 \times 10\%$) for the Egyptian students.	Internet Addiction Test (IAT); Mobile Phone Involvement Questionnaire (MPIQ, 8 items); Epworth Sleepiness Scale (ESS). Statistical analysis: t-test/Mann–Whitney, χ^2/Fisher, Pearson correlation; $\alpha = 0.05$.	• IAT mean: Saudi 76.94 ± 26.27 vs Egypt 55.91 ± 17.49 ($p < .001$); severe IA: 42.69% (Saudi) vs 10.31% (Egypt) ($p < .001$).<br>• MPIQ "highly involved": 41.50% (Saudi) vs 5.45% (Egypt) ($p < .001$). ESS mean: Egypt 17.47 ± 3.99 vs Saudi 16.80 ± 3.83 ($p = .024$).<br>Associations: IA severity ↔ higher ESS (Egypt $p = .002$; Saudi $p < .001$); IA ↔ lower GPA (Egypt $p = .002$; Saudi $p < .001$). MPIQ ↔ GPA: negative and significant in Saudi only ($p < .001$).	Female-only sample; cross-sectional design (no causality); self-report measures; MPIQ does not differentiate mobile vs smartphone; need for app-based, smartphone-specific tools and longitudinal designs.	**Quality Rating Moderate**<br>→ Strengths: two-site comparative design; validated instruments (IAT, MPIQ, ESS); appropriate tests (t-test/χ^2/Pearson).<br>→ Limitations: cross-sectional; female-only; self-report; MPIQ not smartphone-specific.

**8**	**Eskin Bacaksız et al. (2022) [42]**	Turkey	Nomophobia, netlessphobia, and fear of missing out in nursing students: A cross-sectional study in distance education	Descriptive, cross-sectional<br><br>**Aim:** to determine the levels of nomophobia, netlessphobia, and fear of missing out (FoMO) in nursing students during distance education and identify predictors of nomophobia	Country: Turkey<br>Sample size: 802 nursing students from 3 universities<br>Age: Mean = 20.53 ± 1.77, range 17–35<br>Gender: 78.9% female<br>Largest group: 3rd-year students (32.0%)<br>Sampling: Convenience sampling via online survey	Nomophobia Questionnaire (NMP-Q)<br>Netlessphobia Scale<br>Fear of Missing Out Scale (FoMO)<br>Demographic Information Form<br>All instruments validated in Turkish	• Students reported using smartphones 5.13 ± 2.26 hours/day, and internet on phones 4.65 ± 2.74 hours/day.<br>• They scored a mean of 2.98 (±0.78) on the Nomophobia scale, 2.53 (±0.87) on Netlessphobia, and 2.18 (±0.80) on FoMO.<br>• Netlessphobia alone explained 44.4% of the variance in nomophobia, FoMO 18.5%, and both combined 45.4%.<br>• Positive correlations were found between Nomophobia and Netlessphobia ($r = 0.666$) and FoMO ($r = 0.430$), $p < .001$.	Use of self-report data<br>Non-random sampling<br>Cross-sectional design (no causality)<br>Conducted during COVID-19 in a distance learning context	**Quality Rating Moderate**<br>→Strengths: large sample, strong psychometrics, multivariate analysis<br>→Limitations: self-report bias, no control for confounders, non-probabilistic sample
**9**	**Gaber-Hamzaa et al. (2024) [43]**	Egypt	Examining Nursing Students' Prevalence of Nomophobia, and Psychological Alienation and Their Correlates With Fear of Missing Out: A Multisites Survey	Descriptive cross-sectional study<br><br>**Aim:** The study aims to determine the prevalence and levels of nomophobia among nursing students in six universities assess their degree of psychological alienation explore the relationship between nomophobia psychological alienation and fear of missing out and examine whether psychological alienation and fear of	This study was conducted among 1,273 undergraduate nursing students at six Egyptian universities: North Sinai, South Sinai, Port-Said, Suez Canal, Suez, and Damanhur University, Egypt. From June 2023 to November 15, 2023, the students were surveyed using questionnaires on nomophobia, fear of missing out, and psychological alienation.	Nomophobia Questionnaire (NMP-Q)<br>Psychological Alienation Scale<br>Academic FoMOs<br><br>Statistical analysis: Pearson correlations, t-tests, ANOVA	Prevalence:<br>• Moderate nomophobia: 61.4%<br>• Severe nomophobia: 24.7%<br>Psychological alienation:<br>• Moderate: 63.6%<br>• High: 20.6%<br>Associations:<br>Positive correlation between nomophobia and alienation ($r = 0.588$, $p < 0.001$)<br>• Both nomophobia and alienation negatively correlated with academic performance ($r = –0.519$ and $–0.563$, respectively)	-Use of self-reported GPA<br>-Cross-sectional design limits causality<br>-Single-country sample may affect generalizability	**Quality Rating Moderate**<br>→Strengths: validated tools, large sample, correlation analysis<br>→Limitations: no control for confounders, self-reported academic data

				missing out predict nomophobia					
**10**	**Jose et al. (2024) [44]**	India	Impact of problematic mobile phone use among nursing students in India: Exploring associations with depression, insomnia, self-esteem and satisfaction with life	Descriptive cross-sectional study<br><br>**Aim:** to explore the impact of problematic mobile phone use among nursing students in India by examining its associations with depression insomnia self-esteem and life satisfaction	The study, conducted between December 2022 and January 2023, involved 402 Indian undergraduate nursing students (mean age 20.47, range 18–26), 91.3% of whom were female. On average, students owned a personal mobile phone from age 16.8 (±2.4) and began using one at age 12.34 (±4.36).	Problematic Mobile Phone Use Questionnaire (PMPUQ)<br>Academic Stress Scale<br>Pittsburgh Sleep Quality Index (PSQI)<br>Academic performance: self-reported GPA<br><br>Statistical analysis: Pearson correlation, multiple linear regression	Problematic mobile phone use:<br>• Usual users (≤20): 15.9% (n = 64)<br>• Slight addiction (20–49): 45% (n = 181)<br>• Serious problematic use: 39% (n = 157)<br>• Excessive use (>80): 4.5% (n = 18)<br>Mental health indicators:<br>• Depression: 42.5% (n = 171), none severe<br>• Unhappiness with life: 8.9%<br>• Low self-esteem: 70%<br>• Insomnia: 39.6% (n = 159)<br>Associations:<br>Problematic phone use positively correlated with age (r = 0.511, p = 0.002), depression (r = 0.549, p < 0.001), and insomnia (r = 0.360, p < 0.001)<br>Life satisfaction and self-esteem strongly negatively correlated (r = 0.740 and r = 0.785, p < 0.001)<br>Depression predicted higher problematic phone use (β = 0.084, p = 0.080), lower life satisfaction and self-esteem (β = 0.238, p < 0.001), and higher phone addiction (β = 0.107, p = 0.032)	Self-reported academic scores<br>Cross-sectional design limits causal inference<br>No gender stratification or control for confounders	**Quality Rating Moderate**<br>→Strengths: validated tools, regression analysis, relevant outcomes<br>→Limitations: no confounder control, self-reported academic data
**11**	**Kargın et al. (2020) [45]**	Turkey	Evaluation of internet addiction and fear of missing	Descriptive cross-sectional study	Firat University, Faculty of Health Sciences, Turkey;	- Internet Addiction Scale (Young, 1998, Turkish version)	- 3.8% (n=14) of students were pathological internet users<br>- 29.1% (n=107) were at	- Study population limited to one university	**Quality Rating High**<br>→Strengths: large sample,

			out among nursing students	**Aim:** to evaluate the levels of internet addiction and Fear of Missing Out (FoMO) among nursing students	N = 511 nursing students	- FoMO Scale (Przybylski et al., Turkish version)	risk of addiction<br>- Mean IAS score = 42.17 (±19.09)<br>- Mean FoMO score = 23.27 (±8.08)<br>- Positive correlation between internet addiction and FoMO ($r = .332$; $p < .001$)<br>- Males and students aged 21–25 showed significantly higher internet addiction<br>- No significant differences in FoMO scores based on gender, age, or economic status	- Self-report instruments<br>- Cross-sectional design prevents causal inference	validated tools, robust analysis<br>→Limitations: single-site, self-report data, no control for confounders
**12**	**Marletta et al. (2021) [46]**	Italy	Nomophobia in healthcare: an observational study between nurses and students	Cross-sectional observational study.<br><br>**Aim:** to assess levels of nomophobia and to examine its association with device use (daily hours/clinical use) among nursing students.	244 nursing students (1st=79; 2nd=83; 3rd=82) from the Nursing Degree Program, University of Parma; convenience sampling.	NMP-Q (Italian version, Likert 1–7); three ad-hoc items on daily device time, smartphone use during clinical placement, and reasons for consultation.	Device use (students, N=244): >1–2h 6.6%; >2–3h 18.9%; >3–4h 24.2%; >4–5h 22.1%; >5h 28.3%; similar distribution across years (χ^2=5.718; df=8; p=.679). NMP-Q: M=3.52; SD=1.18 (<4); by year: 1st 3.61±1.15; 2nd 3.60±1.08; 3rd 3.36±1.30; ANOVA ns ($F(2,241)$=1.180; p=.31; η^2=0.10). Correlation NMP-Q ↔ hours/day Rs=.287; p<.01.	Self-report questionnaires; no direct observation; timing near COVID-19 (Jan–May 2020) may have influenced behaviour.	**Quality Rating Moderate**<br>→Strengths: clearly described setting/sample; validated outcome; appropriate analyses.<br>→Limitations: non-validated exposures; confounders not pre-specified and not adjusted; convenience sampling; self-report measures; study conducted near the COVID-19 period.
**13**	**Márquez-Hernández et al. (2020) [47]**	Spain	Problematic mobile phone use, nomophobia and decision-making in	Cross-sectional, correlational study<br><br>**Aim:** to examine the relationship between problematic mobile	University of Almería, Spain Sample size: 124 nursing students	Mobile Phone Problem Use Scale (MPPUS) Nomophobia Questionnaire (NMP-Q)	• Nomophobia mean score: 84.12 ± 21.91 (moderate to high)<br>• Strong correlation between problematic	Self-reported data<br>Small, non-random sample	**Quality Rating Moderate**<br>→Strengths: use of validated instruments, significant

			nursing students	phone use, nomophobia, and decision-making styles in nursing students.	Sampling: Convenience sampling<br>Inclusion criteria: ≥18 years old, enrolled in nursing program, not foreign exchange students<br>Mean age: Not consistently reported<br>Gender distribution: Not specified in text	Melbourne Decision Making Questionnaire (MDMQ)<br>Data collected via online questionnaire<br>Analysis: Descriptive statistics, Pearson correlations, multiple regression	phone use and nomophobia (r = 0.703, $p < 0.001$)<br>• Negative correlation between nomophobia and decision-making confidence (r = –0.325, $p < 0.001$)<br>• Positive correlations between nomophobia and avoidant (r = 0.244) and procrastinating (r = 0.311) decision styles<br>• Regression model: problematic phone use and avoidant/procrastinating decision-making styles significantly predicted nomophobia ($p < 0.001$)	Cross-sectional design limits causality<br>Results not generalizable beyond single university	predictive model, relevant correlational analysis<br>→Limitations: small sample, incomplete demographic data, no confounder adjustment, generalizability limited
**14**	**Moreno-Guerrero et al. (2021) [48]**	Spain	Nomophobia and the influence of time to REST among nursing students. A descriptive, correlational and predictive research	Cross-sectional descriptive, correlational, and predictive study<br><br>**Aim:** to determine the level of nomophobia in nursing students and analyze its relationship with rest time (sleep), sociodemographic variables, and technology usage habits.	596 nursing students:<br><br>513 women (86.07%) and 83 men (13.93%), with ages ranging from 18 (17.8%), 19 (54%), 20 (12.4%) to more than 20 years old (15.8%).	Nomophobia Questionnaire (NMP-Q)<br>Self-constructed sociodemographic and behavioral variables (including rest time, screen time, etc.)<br><br>Analysis: Descriptive stats, ANOVA, Pearson correlation, multiple linear regression	Sample profile:<br>Women: 86.07% (n = 513)<br>Men: 13.93% (n = 83)<br>Age distribution: 18 (17.8%), 19 (54%), 20 (12.4%), >20 (15.8%)<br>Nomophobia levels: Intermediate (mean values between 3 and 4)<br>Sleep associations:<br>• Nomophobia positively correlated with various sleep disturbances<br>• Significant links between high nomophobia items (e.g., anxiety without signal, needing access to apps, nervousness without texts/calls) and poor sleep ($p < 0.05$)<br>Regression Model:	Self-report bias<br>Cross-sectional design limits causality<br>Single university sample<br>Non-probabilistic sampling	**Quality Rating High**<br>→Strengths: robust sample, multivariate analysis, validated scale<br>→Limitations: convenience sample, single institution, self-report bias

							• 4 significant predictors explained 9.2% of the variance in hours of smartphone use (Adj. R^2 = 8.6%)<br>• Greater phone use predicted greater negative impact on sleep		
**15**	**Sadeghi et al. (2025).<br>[18]**	Iran	Exploring the prevalence of nomophobia, its contributing factors, and the relationship with social interaction anxiety among nursing students	Cross-sectional, descriptive-analytical study.<br><br>**Aim:** to estimate the prevalence and severity of nomophobia and to examine its relationship with social interaction anxiety in nursing students.	School of Nursing & Midwifery, Kermanshah (Iran); n = 258 nursing students; convenience sampling; mean age 23.1 ± 3.1; 52.7% female.	Nomophobia Questionnaire (NMP-Q); Social Interaction Anxiety Scale (SIAS); demographics and smartphone-use behaviours.	All students (n = 258) showed nomophobia: mild 40.7%, moderate 40.3%, severe 19.0%; social anxiety 25.6%. r = .37 ($p < .001$); $R^2adj \approx .21$ (social interaction anxiety = only independent predictor).	Cross-sectional design; self-report; convenience, single-centre sample; lack of exploration of socio-cultural moderators and long-term outcomes.	**Quality Rating High**<br>→Strengths: validated instruments; clear inclusion criteria and setting/sample description; appropriate statistics.<br>→Limitations: no confounder control; convenience, single-centre sample; self-report → limited generalisability.
**16**	**Tárrega-Piquer et al. (2023)<br>[49]**	Spain	Nomophobia and Its Relationship with Social Anxiety and Procrastination in Nursing Students: An Observational Study	Observational cross-sectional study<br><br>**Aim:** to assess the levels of nomophobia and their relationship with social anxiety and academic procrastination in undergraduate nursing students.	304 nursing students from a Spanish university<br>Mean age: 21.8 years (SD = 4.95)<br>83.6% female<br>Convenience sampling<br>Data collected during the 2021–2022 academic year	Nomophobia Questionnaire (NMP-Q)<br>Social Avoidance and Distress Scale (SADS)<br>Academic Procrastination Scale (EPA)<br>Online survey<br>Analyses: Descriptive stats, Pearson correlation,	Mean NMP-Q score: 67.1 (moderate level of nomophobia)<br>Prevalence:<br>• 59.5% moderate nomophobia<br>• 19.4% severe nomophobia<br>Significant positive correlations between:<br>• Nomophobia and social anxiety (r = 0.24, $p < .001$)	Self-reported data<br>Single university limits generalizability<br>Cross-sectional design: no causal inference	**Quality Rating High**<br>→Strengths validated tools, regression model, multiple predictors explored<br>→Limitations: self-report bias, single-site, cross-sectional

						multiple linear regression	• Nomophobia and procrastination (r = 0.27, p < .001)<br>Multiple regression identified procrastination (β = 0.223) and social anxiety (β = 0.167) as significant predictors of nomophobia (p < .01)<br>Students who used smartphones >5 hours/day had higher nomophobia scores		
**17**	**Tuna et al. (2023) [16]**	Turkey	Touch-operated world of teenagers in the distance education process: A cross-sectional study on nomophobia, netlessphobia and fear of missing out in nursing students	Quantitative, analytical cross-sectional study<br><br>**Aim:** to investigate the relationship between nomophobia, netlessphobia, and fear of missing out (FoMO) among nursing students during distance education.	802 volunteer students at the nursing faculties of three public universities in Istanbul<br>Mean age: 20.53 years (SD = 1.77)<br>Gender: 78.9% female<br>Year of study: Mostly 3rd year (32.0%)	Nomophobia Questionnaire (NMP-Q)<br>Netlessphobia Scale (developed by authors)<br>Fear of Missing Out Scale (FoMO)	Average smartphone use: 5.13 ± 2.26 hours/day<br>Average internet use via smartphone: 4.65 ± 2.74 hours/day<br>Most-used platforms:<br>• WhatsApp: 97.3%<br>• Instagram: 82.2%<br>Mean scores:<br>• Nomophobia: 2.98 ± 0.78<br>• Netlessphobia: 2.53 ± 0.87<br>• FoMO: 2.18 ± 0.80<br>Correlations and Regression:<br>-Netlessphobia accounted for 44.4% of the variance in nomophobia *(r = 0.666; p < .001)*.<br>-FoMO accounted for 18.5% of the variance in nomophobia *(r = 0.430; p < .001)*.<br>-Combined, netlessphobia and FoMO explained 45.4% of the total variance in nomophobia (multiple regression).	Self-report data<br>Non-probabilistic sampling<br>Conducted only in Turkey and limited to nursing students<br>Cannot infer causality due to cross-sectional design	**Quality Rating Moderate**<br>→Strengths: large, multi-site sample; validated instruments; robust correlation and regression analysis<br>→Limitations: no confounder control; self-report and convenience sampling reduce generalizability

18	**Yigit et al. (2024) [7]**	Turkey	The effect of nomophobia levels on nursing students' depression, anxiety and stress levels	Quantitative – Cross-sectional, correlational study<br><br>**Aim**: to determine the relationship between nomophobia and depression, anxiety, and stress levels among nursing students	Sample profile:<br>Nursing students: 544<br>Mean age: 21.10 ± 1.32<br>Nuclear family: 82.4% (n = 448)<br>Income equal to expenses: 71.5% (n = 389)<br>Income less than expenses: 19.5% (n = 106)	Nomophobia Questionnaire (NMP-Q)<br>Depression Anxiety Stress Scale – 21 items (DASS-21)<br>Sociodemographic characteristics form	• Mental health indicators (DASS-21):<br>• Depression: 65.6% (n = 357), mean = 11.96 ± 5.99<br>• Anxiety: 66.2% (n = 360), mean = 10.25 ± 5.84<br>• Stress: 35.8% (n = 195), mean = 11.64 ± 5.70<br>Nomophobia:<br>• Mean score: 102.51 ± 27.06<br>Weak positive correlations with depression (r = 0.299, p < 0.001), anxiety (r = 0.274, p < 0.001), and stress (r = 0.322, p < 0.001)<br>Associations:<br>• Significant difference by gender (p < 0.05)<br>• Higher scores linked to carrying a charger/power bank (p < 0.001), checking phone immediately after waking (p < 0.05), using smartphone before sleep (p < 0.05), and avoiding places without internet (p < 0.05	Cross-sectional design (no causal inference)<br>Data collected via self-report scales (social desirability bias)<br>Single institution, limiting generalizability<br>Potential confounding variables not controlled	**Quality Rating Moderate**<br>→Strengths: large sample, validated scales, robust statistical analysis<br>→Limitations: no control for confounders, single-site
19	**Janatolmakan et al. (2024) [11]**	Iran	Nomophobia: Prevalence, associated factors, and impact on academic performance among nursing students	Quantitative – Cross-sectional, correlational study<br><br>**Aim**: to investigate the prevalence, associated factors, and impact of nomophobia on nursing students' academic performance.	500 nursing students enrolled in the second semester of the academic year 2021-2022 at KUMS.	Nomophobia Questionnaire (NMP-Q)<br>and<br>Sociodemographic characteristics	Prevalence 81.4% (mean NMP-Q = 34.6 ± 16.7 /140; mild level). Non-academic use associated with higher nomophobia (B = 3.22, p = .032). Mean use 3.7 ± 5.8 h/day; 33% ≥5 h/day; mean 53.8 checks/day; 42.5% had 10–20 apps; GPA >15/20 in 86.6%; academic use 46.5%.	Cross-sectional design: this study was only able to establish associations between the nomophobia score and sociodemographic variables. The data collection method relied on	**Quality Rating Moderate**<br>→Strengths: large sample, validated scales, robust statistical analysis<br>→Limitations: no control for confounders, single-site

								self-reporting, which introduces the possibility of response bias.	
